# Designing Cascades of Electron Transfer Processes in Multicomponent Graphene Conjugates

**DOI:** 10.1002/anie.202008820

**Published:** 2020-10-15

**Authors:** Francesca Limosani, Ramandeep Kaur, Antonino Cataldo, Stefano Bellucci, Federico Micciulla, Robertino Zanoni, Angelo Lembo, Bingzhe Wang, Roberto Pizzoferrato, Dirk M. Guldi, Pietro Tagliatesta

**Affiliations:** ^1^ Fusion and Nuclear Department Photonics Micro and Nanostructures Laboratory ENEA Via E. Fermi 45 00044 Frascati Rome Italy; ^2^ Department of Chemical Science and Technologies University of Rome Tor Vergata Via della Ricerca Scientifica 1 00133 Rome Italy; ^3^ Interdisciplinary Center for Molecular Materials Department of Chemistry and Pharmacy Friedrich-Alexander University Erlangen-Nürnberg Egerlandstrasse 3 91058 Erlangen Germany; ^4^ Department of Information Engineering Polytechnic University of Marche Via Brecce Bianche, 1 60131 Ancona Italy; ^5^ INFN- National laboratories of Frascati Via Enrico Fermi 40 00044 Frascati Rome Italy; ^6^ Qi technologies Via Monte D'Oro, 2/a 00040 Pomezia Italy; ^7^ Department of Chemistry University of Rome “La Sapienza” Piazzale Aldo Moro 5 00185 Rome Italy; ^8^ Department of Drug Metabolism and PharmacoKinetic IRBM SpA Via Pontina km 30.600 00071 Pomezia Rome Italy; ^9^ Department of Industrial Engineering University of Rome Tor Vergata Via del Politecnico 1 00133 Rome Italy

**Keywords:** graphene nanoplates, zinc-tetraphenylporphyrin

## Abstract

A novel family of nanocarbon‐based materials was designed, synthesized, and probed within the context of charge‐transfer cascades. We integrated electron‐donating ferrocenes with light‐harvesting/electron‐donating (metallo)porphyrins and electron‐accepting graphene nanoplates (GNP) into multicomponent conjugates. To control the rate of charge flow between the individual building blocks, we bridged them via oligo‐*p*‐phenyleneethynylenes of variable lengths by β‐linkages and the Prato–Maggini reaction. With steady‐state absorption, fluorescence, Raman, and XPS measurements we realized the basic physico‐chemical characterization of the photo‐ and redox‐active components and the multicomponent conjugates. Going beyond this, we performed transient absorption measurements and corroborated by single wavelength and target analyses that the selective (metallo)porphyrin photoexcitation triggers a cascade of charge transfer events, that is, charge separation, charge shift, and charge recombination, to enable the directed charge flow. The net result is a few nanosecond‐lived charge‐separated state featuring a GNP‐delocalized electron and a one‐electron oxidized ferrocenium.

## Graphical abstract

Nanocarbon‐based charge‐transfer cascades were designed by integrating electron‐donating ferrocenes with light‐harvesting/electron‐donating (metallo)porphyrins and electron‐accepting graphene nanoplates. The building blocks were bridged through oligo‐*p*‐phenyleneethynylenes. Physico‐chemical characterization of the multicomponent conjugates revealed a selective photoexcitation triggered cascade of charge transfer events.

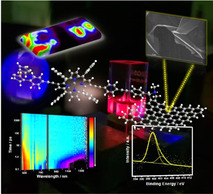

## Introduction

In 2004, graphene moved into the center of attention when Geim and co‐workers isolated for the first time single‐layers of honeycomb networks based on sp^2^‐hybridized carbon atoms by using adhesive tape.^[1]^ Ever since, two‐dimensional graphene turned into the rising star in nanotechnology. All kinds of graphene materials were systematically explored in terms of their characterization and exploited in basic science as well as applications. Leading examples are found in the emerging areas of nanoelectronics, energy‐storage, biosensors, and photoconversion systems.[^2^, ^3^, ^4^, ^5^, ^6^] Graphene gives rise to outstanding electronic and mechanical features; high thermal conductivity^[7]^ and tensile strength just to mention a few.^[8]^ There is reason to believe that this material is ideally suited not only to outperform semiconducting silicon, but also to replace it, for example, in solid‐state devices in the computer and micro‐ and nanoelectronics.

To demonstrate the wider applicability of graphene its poor solubility/dispersibility needs to be overcome. Attractions, which are based on π‐π interactions between individual graphene layers, are the true bottleneck that needs to be tackled. Transformation by means of chemical functionalization based on the use of molecular building blocks is one of the very few high potential approaches toward the manipulation of the band gap in graphene[^9^, ^10^] Among the molecular building blocks light harvesters such as porphyrins, phthalocyanines, etc. stand out. Their utilization allows for a much better overall stability of the respective graphene dispersions. In addition, it affords highly versatile functional materials with photo‐ and redox‐active constituents.

A sound understanding of graphene materials is essential for tuning those electronic communications and interactions that are needed for breakthroughs in technological applications.[^5^, ^11^] Of great importance is the band‐gap design in graphene materials through chemical functionalization/doping to enable non‐linear optics,^[12]^ photovoltaics,^[13]^ energy‐storage,^[14]^ and biosensing.^[15]^ As of today, numerous examples of non‐covalently functionalized graphene‐based electron donor‐acceptor systems exist, and they are well understood.[^16^, ^17^, ^18^] Several chemical methodologies, which are based on well‐established routes, have been developed to modify bulk graphite and, subsequently, to stabilize single and/or few layers of graphene.[^19^, ^20^, ^21^, ^22^] Cycloadditions, free‐radical additions, and click reactions represent those organic reactions that are most frequently used when sp^2^‐carbons of graphene are covalently functionalized. Among the aforementioned, cycloadditions of porphyrins to pristine graphene is the most promising choice.[^23^, ^24^, ^25^, ^26^, ^27^] For example, Prato and co‐workers reported that the 1,3‐dipolar cycloadditions permit covalent functionalization of graphene through a condensation reaction.^[28]^ Aminoacids, on one hand, and carbonylic groups, on the other hand, react by forming iminium salts, which subsequently decarboxylate and yield azomethine ylides. As a consequence, fused pyrrolidines are incorporated onto the basal plane of graphene. In principle, multiple additions facilitate the introduction of several functional constituents in a single reaction step. Notably, this procedure has been extensively exploited for the chemical modification of fullerenes, nanodiamonds,^[29]^ carbon nanotubes^[30]^ and carbon nanohorns,^[31]^ with applications in various fields. In stark contrast, acylations are preferentially carried out with the oxygen‐containing groups on graphene edges.

Considering the large‐scale applications of few‐layered graphene‐based materials we have investigated for the first time the functionalization of a new carbon‐based material: graphene nanoplates (GNP). GNP is best described as few layers of graphene, which is produced in high‐yields in research‐based laboratories. The design of graphene‐based functional materials for charge‐transfer applications requires suitable methods and choices of redox‐ and photo‐active constituents.

What still remains as an open question is what causes the short lifetime of radical ion pair states upon either electron or hole injection into exfoliated graphene and/or few layers graphene.^[16]^ In the case of few layers graphene, to which (metallo)phthalocyanines are linked in the form of either electron donors or acceptors, the lifetimes are limited to a few hundred picoseconds. The only approach that assists in addressing this question is to control the electron donor‐acceptor distance. To the best of our knowledge, (metallo)porphyrins have never been used in combination with a secondary electron donor to functionalize few layers of graphenes. Setting up a versatile redox gradient along chains of multiple electron donor‐acceptor enables cascades of short‐range charge transfer events and, in turn, ensures the spatial separation of charges, that is, one‐electron reduced and one‐electron oxidized electron donors. In terms of molecular building blocks, we decided to explore (metallo)porphyrins and ferrocenes conjugates owing to their different strength of electron‐donation. This approach is similar to that we explored in fullerene‐based electron donor‐acceptor conjugates, which exhibit long‐lived charge‐separated states as the product of a cascade of several charge‐transfer events.^[32]^ In particular, we focused on the synthesis of β‐modified porphyrins, which feature electron‐accepting C_60_[^32^, ^33^, ^34^] and/or electron‐donating ferrocenes at the β‐, *meso*‐, or 4‐phenyl positions.[^35^, ^36^, ^37^, ^38^, ^39^, ^40^, ^41^] Our past work provided fundamental insights into the unambiguous identification of all the different species that evolve as products of any charge‐transfer reactions, namely charge separation, charge shift, charge recombination, etc.

In light of our experience,[^42^, ^44^] we opted in this work for linking electron‐donating ferrocenes (Fc) with light‐harvesting/electron‐donating (metallo)porphyrins (ZnP) and electron‐accepting graphene nanoplates (GNP) via oligo‐*p*‐phenyleneethynylenes of variable lengths. A unique feature of our nanocarbon‐based materials is the β‐linkage at the (metallo)porphyrins to modulate the electron donor‐acceptor interactions.

## Results and Discussion

### Synthesis

**Figure 1:**
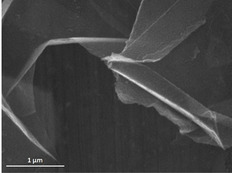
SEM image of graphene nanoplates (GNP).

The synthesis of GNP (Figure 1) was based on a low cost, fast, scalable, and stable fabrication method. It relies on a standard 800 W household microwave (MW) oven and starts with an Asbury® Expandable graphite sample, where the graphene planes were intercalated with chemical substances such as sulphates and nitrates.

After MW irradiation, the samples are best described by a worm‐like morphology with a very large particle area. Using a short ultrasound treatment in isopropylic alcohol, the worm‐like overall structures were removed from GNPs. Correspondingly, the 2D particles with lateral dimensions of tens of micrometers and thicknesses <5 nm correspond to several layers of graphene.

The scanning electron microscope micrograph in Figure 1 confirmed that the intercalated multilayer graphite had undergone expansion.[^45^, ^46^] In fact, even if in the SEM microscope the electrons have typically a low energy of up to 30 keV. As such, nanometer thicknesses and the low atomic weights of carbon allowed electrons to pass, which rendered the underlying plates discernable.

**Scheme 1:**
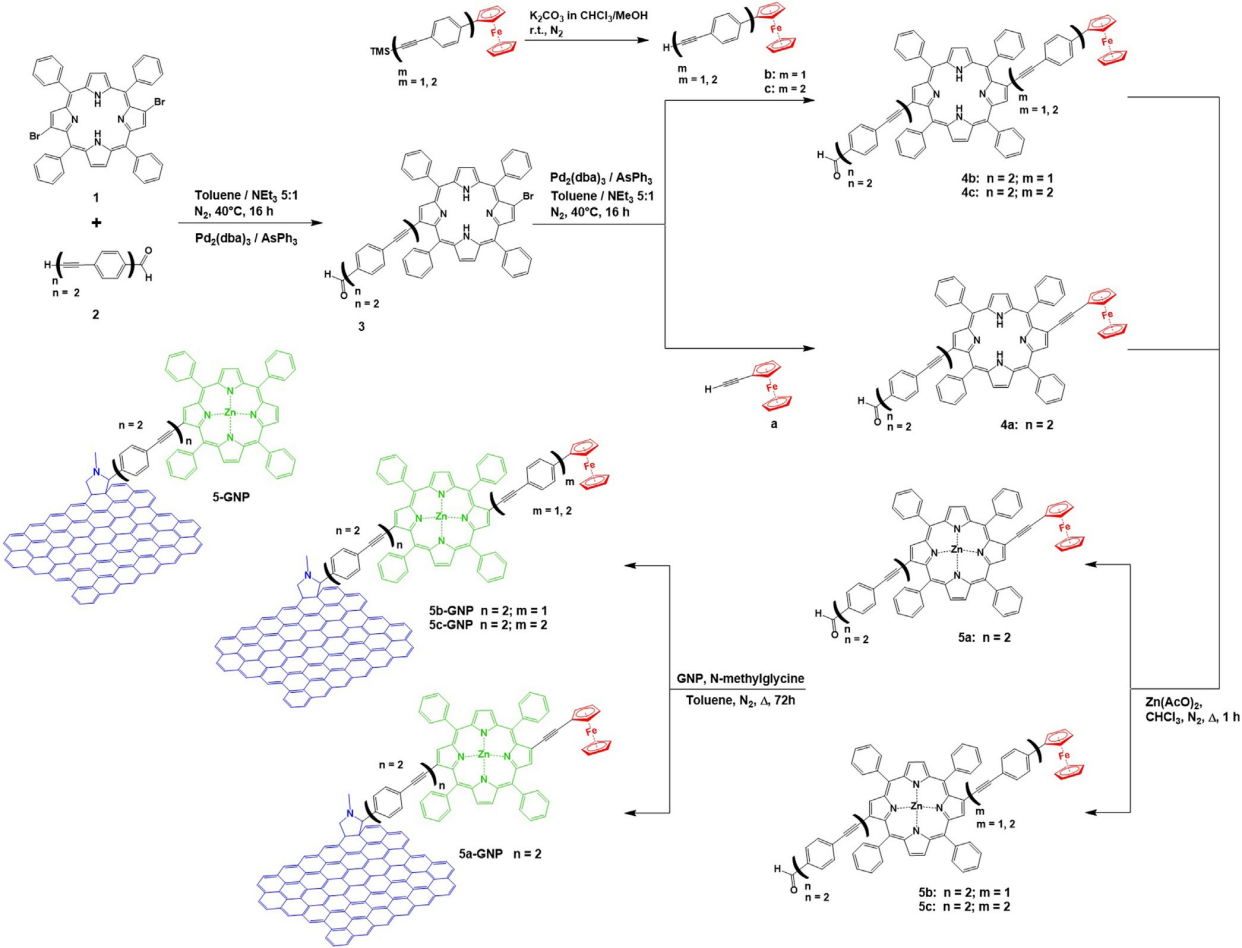
Synthetic pathway towards the new GNP‐conjugates.

In Scheme 1, we summarize the synthetic strategy for obtaining the new GNP‐conjugates. The first step involved the Sonogashira coupling of **1** (H_2_Br_2_) with 1.5 equivalents of 4‐[(4′‐ethynyl)phenyl]‐ethynylbenzaldehyde **2**, affording porphyrin derivative **3** in 36 % yield after chromatographic purification.^[33]^ The stereochemistry of 2,12‐dibromo‐TPP (H_2_Br_2_) as starting material was recently elucidated.^[47]^ The next step was a second Sonogashira coupling reaction between **3** and two equivalents of three different electron donors, namely **a**–**c**, yielding the three different intermediates **4 a**,^[34]^ **4 b**,^[44]^ and **4 c**, in which the ferrocenes are linked to porphyrin by different spacers. By adopting a literature procedure, Zn^II^ ion was inserted in the cavity of the porphyrin to give **5 a**, **5 b**,^[33]^ and **5 c** in high yields. In the last step, the Prato‐Maggini reaction^[28]^ was used to connect GNP to **5(a–c)**, and **5(a–c)‐GNP** were isolated. Each step of the reaction was characterized and confirmed using Mass spectrometry and NMR spectroscopy as reported in SI (from Figure S4 to Figure S9).

### X‐Ray Photoelectron Spectroscopy

**Figure 2:**
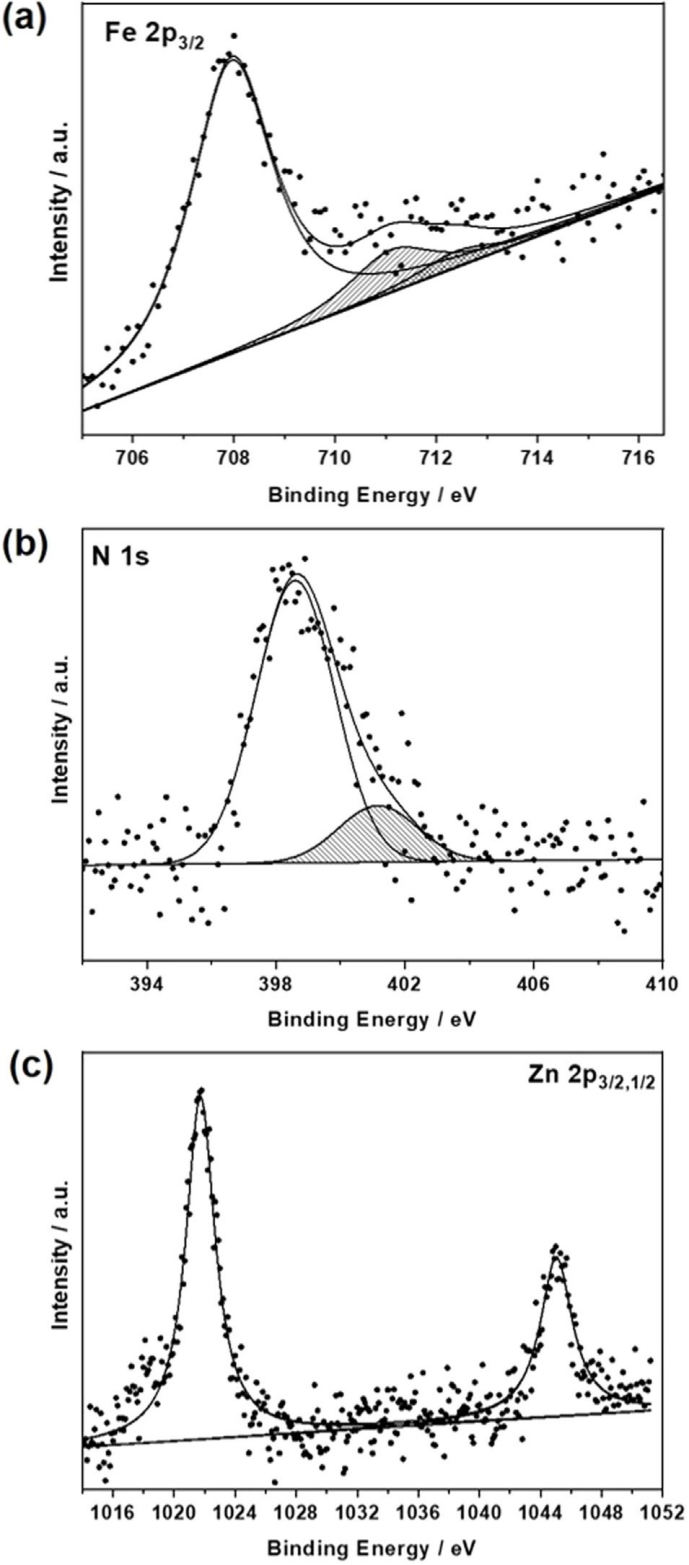
XPS spectra of **5 b‐GNP**: a) Fe 2p_3/2,_ b) N 1s, and c) Zn 2p_3/2,1/2_ photoemission regions with experimental data and peak‐fits as dots and lines, respectively.

XPS analysis was performed with GNP before and after its functionalization to establish the differences between **5 a**, **5 b**, and **5 c**, on one hand, and **5 a‐GNP**, **5 b‐GNP**, and **5 c‐GNP**, on the other. An example is given in Figure 2, in which the Fe 2p_3/2_ and N 1s peaks are surveyed for **5 b‐GNP**. In the Fe 2p photoemission region a major peak evolves at 708.0 eV next to a low‐intensity feature at 711.1 eV and a satellite line at higher energies. In agreement with literature reports,^[48]^ the Fe 2p main peak is assigned to Fe^II^, while the other two components originate from Fe^III^. The co‐existence of Fe^III^ and Fe^II^ is likely to be due to interactions of the ferrocene valence orbitals with the conjugated network of π‐orbitals in graphene. As a matter of fact, some of us have reported on a redox‐active Si (100) hybrid system, to which fullerenes were covalently functionalized.^[48]^ In the present study, XPS spectra of Fe 2p revealed the presence of Fe^III^ and Fe^II^ in a different ratio with respect to the substituted ferrocenes directly bound to the same Si (100) via C=C or C≡C anchors. The bonding between the Fc‐ZnP and GNP fragments was corroborated by the presence of two distinct N 1s peaks, that is, porphyrin nitrogens (398.6 eV) and substituted amine groups (401.1 eV). From Figure 2 b, a 4:1 ratio is described. The aforementioned results were expected based on linking **5 b** to **GNP** in **5 b‐GNP**.

By virtue of an N/Fe atomic ratio, which is 5.0, and a Zn/Fe atomic ratio, which is 1.0, we confirm the intact nature of the attached Fc‐ZnP fragments. Figure 2 c displays the Zn 2p_3/2,1/2_ photoemission region, which reveals a peak at 1022.4 eV, and, which confirms the presence of the (metallo)porphyrin.

Results for **5 a‐GNP** (Figure S10) and **5 c‐GNP** (Figure S11), which are summarized in the SI, attest the linkage of **5 a** and **5 c**, respectively, to **GNP. 5‐GNP** (Figure S3), which was also characterized by XPS (Figure S12), was used as a reference in the transient absorption spectroscopy measurements.

### Raman Spectroscopy

**Figure 3:**
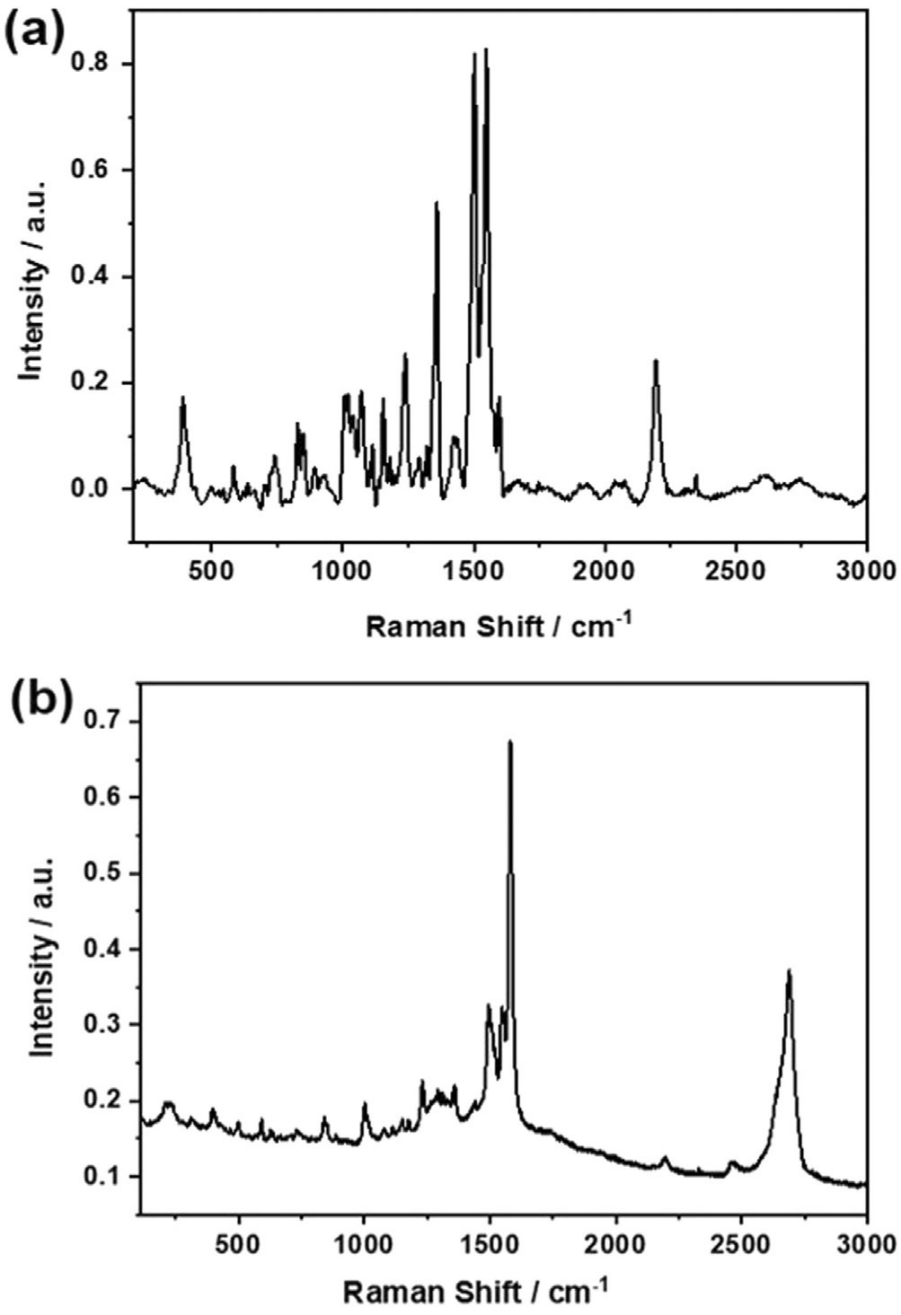
Raman spectra, using a 600 l mm^−1^ grating, in the 200–3000 cm^−1^ spectral range of a) **5 a** and b) **5 a‐GNP**.

To validate the GNP functionalization with the different Fc‐ZnP fragments, Raman spectra were compared. The Raman spectrum of **5 a** is dominated by the ZnP‐centered fluorescence. Consequently, a correction of the Raman spectrum, by subtraction of the fluorescence, was performed. This is shown in Figure 3. Based on the ZnP fluorescence quenching in the conjugates,^[49]^ a well‐resolved Raman spectrum evolves for **5 a‐GNP**. The most intense peaks are attributed to graphene. For example, the G‐ and 2D‐bands are discernable at 1580 and 2700 cm^−1^ respectively. Additional peaks are seen at 852, 1001, 1234, 1492, and 1551 cm^−1^ and they all relate to ZnP (Figure 3 b). When turning to **5 c‐GNP** (Figure S13), some subtle changes relative to **5 a‐GNP** are evident. ZnP‐centered peaks at 403, 592, and 1000 cm^−1^ are noticeably enhanced.

**Figure 4:**
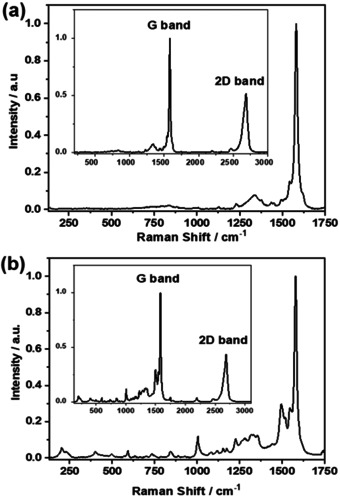
Raman spectra, using a 1800 l mm^−1^ grating, acquired with a 633 nm laser. Comparison of a) **GNP** mixed with **5 b** and b) **5 b‐GNP** conjugate in the 200–1700 cm^−1^ spectral rang e. In the inset, the spectra in the 200–3000 cm^−1^ spectral range show G‐ and 2D‐bands.

To testify that the Fc‐ZnP fragments are covalently linked in the conjugates to GNP rather than adsorbed, comparative assays were performed. A leading example is gathered in Figure 4, where a comparison between **5 b‐GNP** and a mixture of **GNP** and **5 b** is shown. The Raman peaks at 1540 and 2200 cm^−1^, which are diagnostic probes for the presence of Fc‐ZnP fragments, are present in both cases. But, any other peaks, related to the covalent bonding, are absent in the mixtures of **GNP** and **5 b** (Figure 4 a). Peaks at 200, 300, 400, and 600 cm^−1^ (Figure 4 b) are attributed to **5 b** vibrations. More important are, however, the peaks at 235 and 400 cm^−1^ as well as the intensity inversion of the peaks at 590 and 630 cm^−1^. All of them testify the covalent bonds between **5 b** and **GNP**. A spectral deconvolution implies that the peaks between 1250 and 1400 cm^−1^ are due to the superimposition of the GNP‐centered D‐band at around 1350 cm^−1^, and **5 b** vibrations around 1250 and 1400 cm^−1^. Please note that all of the peaks are well defined in the spectrum of the conjugate. When turning to the spectrum of the **GNP** and **5 b** mixtures, the GNP‐centered D‐band dominates, while those peaks, which are due to the Fc‐ZnP fragments, are missing. Most important are the differences in the 1500–1600 cm^−1^ range. An intensification of the 1500 cm^−1^ peak highlights the graphene‐porphyrin linkage. The presence of the 1560 cm^−1^ shoulder suggests the formation of the five‐membered ring obtained during the Prato‐Maggini reaction.^[50]^

**Figure 5:**
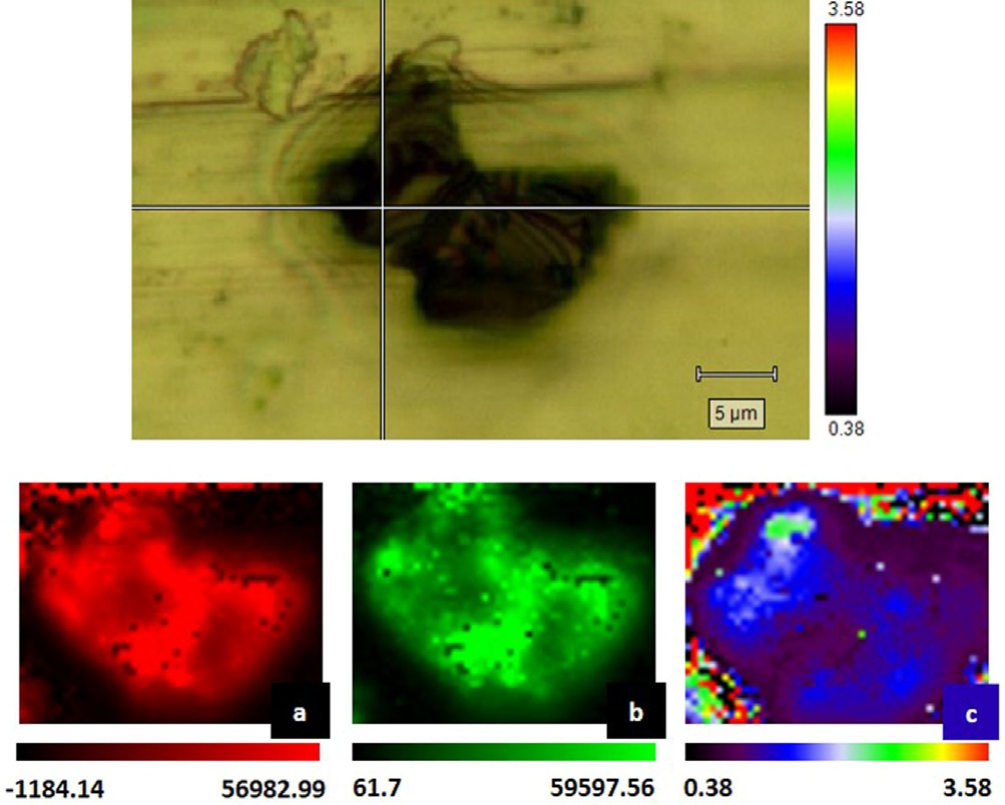
Raman spectral imaging of **5 c‐GNP**: a) peak area of the G‐band at 1579 cm^−1^, b) peak area of **5 c** at 1498 cm^−1^, c) peak area of the G‐band/peak area of **5 c**. The final map is obtained with a sampling step of 100 nm.

To localize the binding sites between the **GNP** and Fc‐ZnP fragments, Raman spectral imaging was carried out. The results for **5 c‐GNP** are reported in Figure 5. In details, the G‐band at 1579 cm^−1^ was used to map the GNP flakes (Figure 5 a), while the peak at 1498 cm^−1^ to identify the presence of **5 c** (Figure 5 b). On the GNP, the localization of unfunctionalized sites versus functionalized ones was concluded from the ratio of the peak area of the G‐band at 1579 cm^−1^ relative to the peak area of **5 c** at 1498 cm^−1^.

The localization of the Fc‐ZnP fragments, shown in Figure S13, was derived by analyses of the peak areas at 1498 cm^−1^. Higher values were correlated with the presence of the fragments at a specific site. As shown in Figure 5 c, the images obtained for **5 c‐GNP** through spectroscopic data highlight the homogeneous presence of Fc‐ZnP fragments (Figure 5 b) on the GNP surface (Figure 5 a), confirmed by the ratio of peak areas.

On the contrary, the distribution of Fc‐ZnP fragments on GNP in **5 a‐GNP** is by far more heterogeneous (Figure S15). The corresponding spectra for **5 b‐GNP** are displayed in Figure S14.

### Atomic Force Microscopy

As a complement, atomic force microscopy (Figures S16–19) was performed with samples that were drop casted from THF dispersions onto clean silicon wafers. For all GNP‐conjugates, next to residues stemming from solvents and Fc‐ZnP fragments, graphene flakes with an average height of 5–10 nm are present. This is, however, more than what we observed just for **GNP**. We attribute this finding to the porphyrins and the phenyl‐ethynyls, which are likely to result into stronger stacking. All in all, the results indicate that the conjugates consists of multi‐layered turbostratic graphene when studied in the solid state.

### Steady‐State Absorption and Fluorescence Spectroscopy

Various references were used in the following sections: commercially available zinc‐tetraphenylporphyrin(**ZnTPP**), Fc‐ZnP based conjugates (**HZnPa**, **HZnPb**, (Figure S1)),^[52]^ a β‐substituted zinc porphyrin‐based reference (**β‐ZnP‐ref**)^[32]^ (Figure S2), and **5‐GNP** (Figure S3).

**Figure 6:**
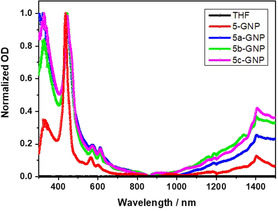
Absorption spectra of **5‐GNP** (red), **5 a‐GNP** (blue), **5 b‐GNP** (green), and **5 c‐GNP** (magenta) recorded in THF under ambient conditions.

Figures 6, S20, and S22 show the absorption spectra of the different GNP conjugates together with the **ZnTPP** and **β‐ZnP‐ref** references, respectively. Overall, β‐substitution has a considerable influence on the absorption features, which include broadening and red‐shifts relative to **ZnTPP**. Notably, the Soret‐band absorption shifts from 427 nm in **ZnTPP** to 433 nm in **5‐GNP**, and 445 nm in **β‐ZnP‐ref**, **5 a‐GNP**, **5 b‐GNP**, and **5 c‐GNP**. Likewise, the Soret‐band absorptions split. Also, the ratio of the Q(0,0) to Q(1,0) band absorption is changed from 0.38 in **ZnTPP** to 0.69, 0.80, and 1.0 for **5‐GNP**, **5 a‐GNP**, and **5 b**/**5 c‐GNP** respectively. This is indicative for the break in degeneracy of the ^1^E(a_2u_, e_g_) and E(a_1u_, e_g_) levels. Contributions stemming from GNP are discernable throughout the entire 300 to 1500 nm range implying an overall increase in the optical density, which is particularly strong in the blue region of the solar spectrum pertaining to the π‐π* transitions. In the near‐infrared NIR region, the plasmonic resonances of graphene are also visible. Fc‐centered absorptions are invisible to us due to the low extinction coefficients.

From the absorption data, the band‐gap of the GNP was determined by means of the Tauc analysis (see for details the SI). The direct band‐gap is 3.1 eV, which reflects the semi‐conducting nature of GNP (Figures S20, S21). By employing the Tauc analysis for the GNP absorption we observed a narrowing of the band‐gap due to functionalization, with values in the range of 2.60–2.75 eV (Figure S23).[^53^, ^54^, ^55^, ^56^]

To this end, the absorption spectra of **5 a‐C_60_**,^[32]^ **5 a‐GNP**, and **ZnTPP** (Figure S22b) give rise to subtle changes in the form of broadened and red‐shifted absorption bands. All the changes in the electronic properties indicate that the molecular orbitals of ZnP are subjected to perturbation due to the steric effects and β‐substitution.

**Figure 7:**
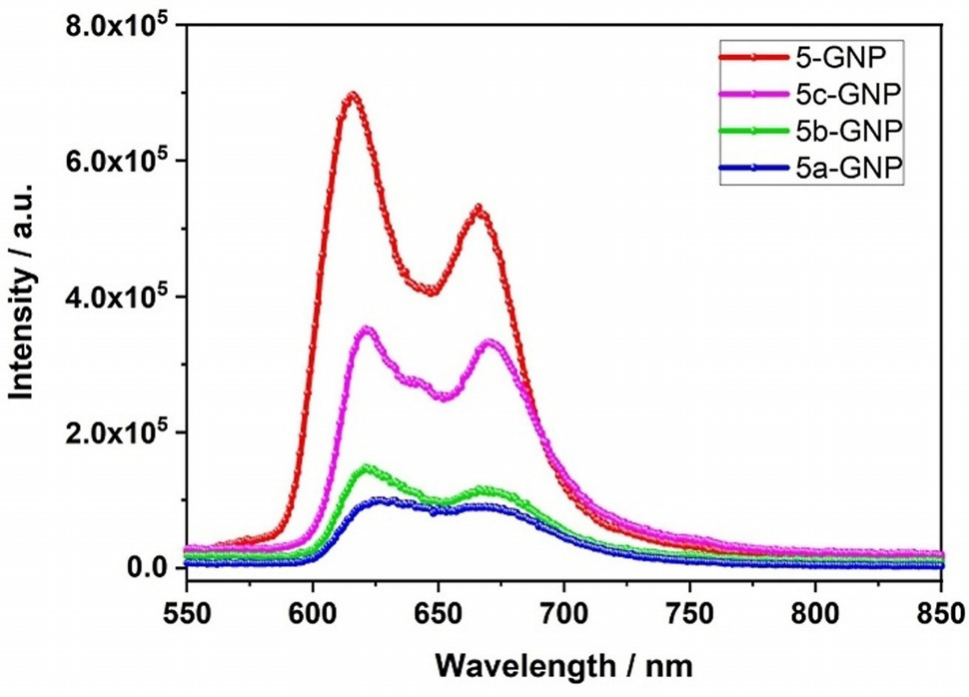
Fluorescence spectra of **5‐GNP** (red), **5 c‐GNP** (magenta), **5 b‐GNP** (green), and **5 a‐GNP** (blue) recorded in THF under ambient conditions upon 445 nm excitation.

Probing the excited‐state interactions between the (metallo)porphyrins and GNP was realized by recording the fluorescence of **β‐ZnP‐ref** and **ZnTPP** as references and compared to that of **5‐GNP**, **5 a‐GNP**, **5 b‐GNP**, and **5 c‐GNP**—Figures 7, S25, and S26a, b. At first glance, the fluorescence spectra of **5 a‐GNP**, **5 b‐GNP**, and **5 c‐GNP** resemble that seen for **ZnTPP**. However, a notable red‐shift of ≈20 nm confirms the more extended π‐conjugation.[^32^, ^34^, ^44^] A closer look reveals a distance‐dependent trend when comparing the ZnP fluorescence as a function of separation between the electron donating Fc and ZnP (Figure 7). For instance, **5‐GNP**, which lacks any Fc, exhibits a minimum of fluorescence quenching. This increase, however, in the following order: **5 c‐GNP**<**5 b‐GNP**<**5 a‐GNP**. This trend clearly supports the notion that the addition of Fc as a second electron donor promotes an additional excited state deactivation. A likely rationale is a charge transfer, whose efficiency decreases with distance. In this context it is important to note that molecular orbital theory has underlined that the unique π‐electron system of graphene acts as an electron acceptor when directly linked to a (metallo)porphyrin.^[51]^ π‐Stacking between electron‐abundant aromatics and carbon materials is yet another cause for the fluorescence quenching.

### Electrochemical Studies

To shed light on the electrochemical behavior of **5 a‐GNP**, **5 b‐GNP**, and **5 c‐GNP**, cyclic voltammetry studies were carried out in acetonitrile. The GCEs were modified prior to the any of the measurements by drop casting the sample solutions (Scheme S1).

In Figure S27, the CVs of the Fc‐ZnP fragments **5 a**, **5 b**, and **5 c**, are displayed, as they assist in understanding the interactions between Fc, ZnP, and GNP. From Figure S27a, which shows the cyclic voltammogram of **5 a**, we deduce a first, quasi‐reversible oxidation at +0.69 V. This is Fc‐centered. In addition, a second, irreversible oxidation is seen at +0.90 V, which is attributed to the ZnP oxidation. In terms of reduction, two irreversible steps are seen for **5 a** at −1.27 and −1.54 V. Both of them are ZnP‐centered. **5 b** (Figure S27b) reveals a first, quasi‐reversible oxidation at +0.59 V followed by a second, irreversible oxidation at +0.87 V. These are Fc‐ and ZnP‐centered, respectively. The reduction is characterized by a first, reversible step at −1.28 V and a second, irreversible step at more negative values. For **5 c**, a reversible oxidation at +0.54 V is accompanied by two irreversible oxidations at +0.96 and +1.24 V and two irreversible reductions at −1.34 and −1.54 V, respectively (Figure S27c). Looking at the Fc‐centered oxidation in **5 a**, **5 b**, and **5 c** we derive a notable distance dependence. In other words, the closer Fc is placed to ZnP, the stronger the shift to more positive oxidations is.

To study the interactions between Fc, ZnP, and GNP, CV measurements were also performed with **5 a‐GNP**, **5 b‐GNP**, and **5 c‐GNP**. They are shown in Figure S28 and summarized in Table 1. Notable shifts are seen for **5 a‐GNP**, **5 b‐GNP**, and **5 c‐GNP** relative to **5 a**, **5 b**, and **5 c**. With the help of the electrochemical and the steady‐state absorption/ fluorescence spectroscopy we employed the following equation,

(1) $${\Delta G}_{CS}^{{}^{\circ }}={[e(E}^{ox}\left(D/D^{•+}\right)-E^{red}\left(A/A^{•-}\right))]-E_{00\;}\left(ZnP\right)$$

**Table 1:** Redox potentials of the Fc‐ZnP fragments and GNP‐conjugates recorded in argon‐saturated acetonitrile with TBAPF_6_ as supporting electrolyte, Ag/Ag^+^ as reference electrode, Pt as counter electrode, and glassy carbon as working electrode, at a scan rate of 0.01 Vs^−1^

Compound	Oxidation			Reduction	
	I	II	III	I	II
**5 a**	+0.69	+0.90	–	−1.27	−1.54
**5 b**	+0.57	+0.88	–	−1.28	−1.53
**5 c**	+0.54	+0.96	+1.24	−1.34	−1.54
**5 a‐GNP**	+0.51	+0.94	–	−1.33	−1.53
**5 b‐GNP**	+0.56	+0.90	+1.15	−1.31	–
**5 c‐GNP**	+0.56	+0.88	–	−0.50	−1.47

to corroborate that the formation of Fc^.+^‐ZnP^.−^‐GNP is exergonic by ca. −0.2 eV relative to the energy of the ZnP first singlet excited state with 2.0 eV.[^32^, ^57^] To evaluate the energetics of those charge‐separated states which are based on the one electron reduced form of GNP, that is, Fc‐ZnP^.+^‐GNP^.−^ and Fc^.+^‐ZnP‐GNP^.−^, we referred to the values reported in the literature for the reduction of graphene sheets. After correcting the reported values versus Ag/Ag^+^, the reduction was determined to be in the range from −0.5 to −0.8 V.[^58^, ^59^] Interestingly, this value matches well with the reduction observed for **5 c‐GNP** at −0.5 V. Therefore, formation of Fc^.+^‐ZnP‐GNP^.−^ (≈1.2 V) by means of a charge shift from Fc^.+^‐ZnP^.−^‐GNP (1.86 V) and Fc‐ZnP^.+^‐GNP^.−^ (≈1.55 V) is exergonic by at least −0.3 eV.

### Transient Absorption Spectroscopy

To further elaborate on the excited state interactions, which were concluded from the steady‐state fluorescence assays we performed transient absorption measurements and used an excitation wavelength of 460 nm. To interpret the dynamics in the reference as well as conjugates, the raw data were analyzed using multi‐wavelength analyses and global target analyses.^[60]^ The details are given in the experimental section.

Firstly, we looked at **β‐ZnP‐ref**. Upon excitation, singlet excited state features in the visible and the near‐IR‐regions evolve at around 500, 598, 620, and 1367 nm, next to the ground state bleaching at 578 and 618 nm. Based on multi‐wavelength and the global analyses we gathered the best fit by employing a three species kinetic model (Figure S30a), which is in sound agreement with the deactivation model known for **ZnTPP**. Following Soret‐band absorption excitation, the second singlet excited (^1**^ZnP) undergoes internal conversion to afford the first singlet excited state (^1*^ZnP) (SAS1) on an ultrafast fs‐time scale, which we were unable to resolve. ^1*^ZnP is then subjected to intersystem crossing within 1.0 ns after the vibrational relaxation (^1*^ZnP_rel_) (SAS2) in 36.0 ps to generate the long‐lived triplet excited state (^3*^ZnP) (SAS3) with characteristic transients at 498 and 920 nm (Figures S29 and S30).

Next, we turned to the GNP‐conjugates. We looked first at **5‐GNP** as it provides important insights into understanding the essential interactions between (metallo)porphyrin and GNP and the dynamics thereof (Figure S31).^[61]^ Relative to **β‐ZnP‐ref**, the **5‐GNP** conjugate exhibits upon 460 nm excitation not just the ^1*^ZnP‐related features in the visible region, but also the GNP‐centered phonon bleaching in the near‐infrared region as SAS1. It is within 9.90 ps that SAS2 is formed, whose transient spectrum is positive and spans across the visible and the near‐infrared regions. Of particular importance is the broad maximum, which ranges from 610 to 670 nm, and the positive transient in the near‐infrared region. All of this resembles what has been summarized in the literature as the one‐electron oxidized form of ZnP, on one hand, and the one‐electron reduced form of GNP: ZnP^.+^‐GNP^.−^.^[5]^ The latter state charge recombines to afford the energetically lower lying ^3*^ZnP as SAS3. 550 ps is the time that it takes for charge recombination (Figures S32 and S33).

**Figure 8:**
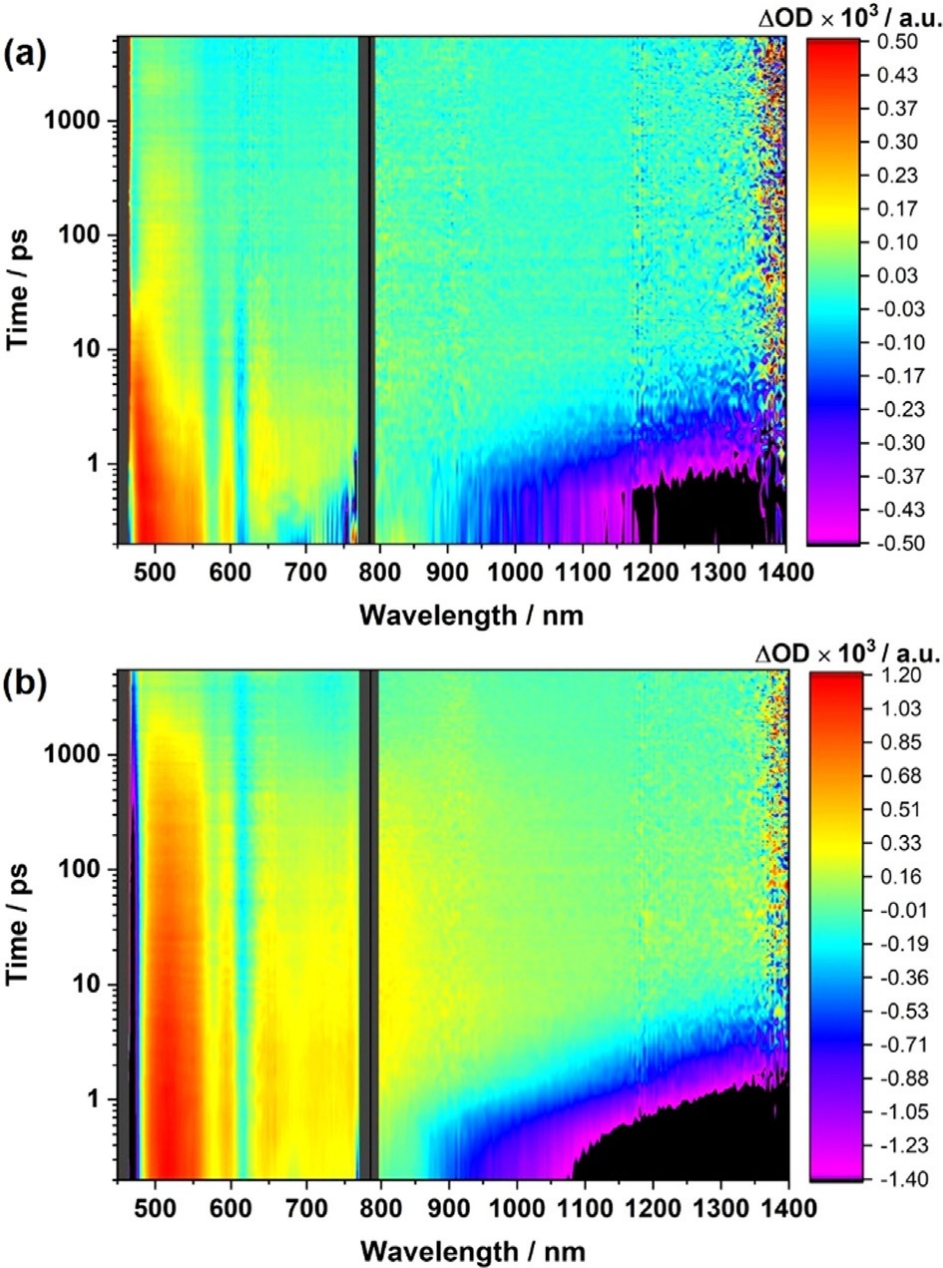
Fs‐transient absorption spectra of a) **5 a‐GNP**, and b) **5 c‐GNP**, recorded with several time delays between 0.1 and 5500 ps in the visible and near‐infrared region (*λ*_exc_=460 nm) in argon‐saturated THF at room temperature.

The picture changes quite a bit, when probing **5 a‐GNP**, **5 b‐GNP**, and **5 c‐GNP**. Concerning the conjugates, ZnP is excited and affords the second singlet excited state. It is the latter that is prone to undergo ultrafast internal conversion to yield the first singlet excited state. Upon excitation, the ^1*^ZnP singlet excited state with its characteristic maxima and minima in the visible region is directly formed, along with the GNP phonon bleaching in the NIR region was observed. Considering the redox gradient, which we corroborated in the electrochemical studies, we adopted a kinetic model from the previous work on electron accepting C_60_ linked to ZnP and Fc (Figures S34 and S35).^[32]^ As a matter of fact, the model gave rise to a 100 % convergence at a maximum of 40 iterations to fit the data qualitatively. Starting with the **5 a‐GNP** (Figure 8 a) in which Fc experiences the shortest separation relative to ZnP, we observe that (^1*^ZnP) (SAS1) undergoes in 3.0 ps a parallel decay to generate Fc^.+^‐ZnP^.−^‐GNP and Fc‐ZnP^.+^‐GNP^.−^ as SAS2 and SAS3, respectively.

The characteristic features of both charge transfer products, that is, Fc^.+^‐ZnP^.−^‐GNP and Fc‐ZnP^.+^‐GNP^.−^, compare quite well with the spectroelectrochemical reduction and oxidation of the **β‐ZnP‐ref** (Figure S36). Likewise, the deconvoluted species for the ZnP‐Fc fragment (Figures S38, and S40) match well.^[32]^ A comparison of SAS2 for **5 a‐GNP** in terms of Fc^.+^‐ZnP^.−^‐GNP with that for the species associated spectra deconvoluted in the ZnP‐Fc conjugates reveals a remarkable resemblance (Figure S43a). Detection of the spectroscopic fingerprints of the one‐electron oxidized form of Fc are, however, hampered by the low extinction coefficients.

**Figure 9:**
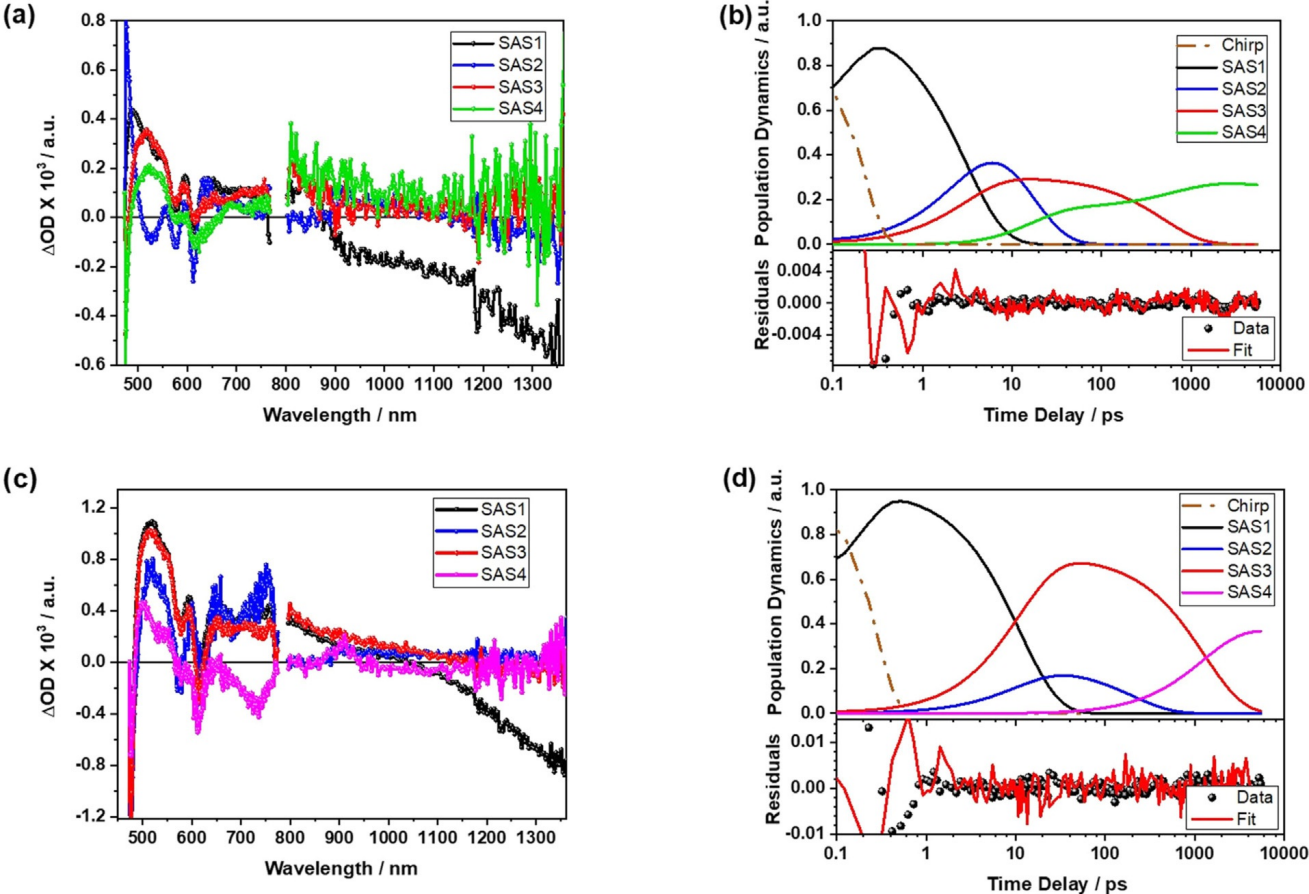
a) Species associated spectra (SAS) of **5 a‐GNP** with Fc‐^1*^ZnP‐GNP (SAS1 in black), Fc^.+^‐ZnP^.−^‐GNP (SAS2 in blue), Fc‐ZnP^.+^‐GNP^.−^ (SAS3 in red), and ^3*^ZnP/ Fc^.+^‐ZnP‐GNP^.−^ (SAS4 in green), deconvoluted using the global‐target analysis of the transient absorption data recorded at an excitation wavelength of 460 nm in THF under ambient conditions. b) Population dynamics of the SAS1 (τ =3.0 ps; CS1=1.8×10^11^ s^−1^ and CS2=9.88×10^10^ s^−1^ ), SAS2 (τ =15.5 ps; CR2=4.5×10^10^ s^−1^ and CSh1=1.93×10^10^ s^−1^), SAS3 (τ =450 ps; CR3=5.55×10^8^ s^−1^), and SAS4 (τ >10.0 ns), along with the residuals of the fits. c) Species associated spectra (SAS) of **5 c‐GNP** with Fc‐^1*^ZnP‐GNP (SAS1 in black), Fc^.+^‐ZnP^.−^‐GNP (SAS2 in blue), Fc‐ZnP^.+^‐GNP^.−^ (SAS3 in red), and ^3*^ZnP (SAS4 in magenta), deconvoluted using the global‐target analysis of the transient absorption data recorded at an excitation wavelength of 460 nm in THF under ambient conditions. d) Population dynamics of the SAS1 (τ =11.0 ps; CS1=1.6×10^10^ s^−1^ and CS2=7.20×10^10^ s^−1^), SAS2 (τ =200 ps; CR2=5.0×10^9^ s^−1^ ), SAS3 (τ =1.20 ns; CR3=3.87×10^8^ s^−1,^ CR3 into the SAS4=4.50×10^8^ s^−1^), and SAS4 (τ >10.0 ns), along with the residuals of the fits.

Next, we note the independent decays of SAS2 and SAS3 with 15.5 and 450 ps, respectively. Overall, decays compete with each other: charge recombination to directly recover the ground state, charge recombination to afford ^3*^ZnP, and charge‐shift to generate Fc^.+^‐ZnP‐GNP^.−^ (Figure 9 a and b). We postulate a charge‐shift predominantly from Fc^.+^‐ZnP^.−^‐GNP with some additional contribution from Fc‐ZnP^.+^‐GNP^.−^, as its lifetime is shortened due to the competing processes that are absent in **5‐GNP**.

For **5 b‐GNP** (Figures S41, S42) and **5 c‐GNP**, (Figures 9 c,d, and S35), which both feature larger separations between the Fcs and ZnPs due to the presence of either one or two phenyl‐ethynyl subunits, the ^1*^ZnP lifetime (SAS1) increases consecutively from 4.0 to 11.0 ps. Following the population from SAS1, the lifetimes of the Fc^.+^‐ZnP^.−^‐GNP (SAS2) charge recombination is fit with 38.5 ps for **5 b‐GNP**, and 200 ps for **5 c‐GNP**. On the other hand, Fc‐ZnP^.+^‐GNP^.−^ as SAS3 is subject in **5 b‐GNP** to a charge recombination within 550 ps. It is, however, the ground state recovery and the triplet state population, which competes with the charge shift, to afford Fc^.+^‐ZnP‐GNP^.−^ as SAS4. SAS4 decays with a lifetime longer than the instrumental timescale. Additionally, SAS4 exhibits contributions from the coexisting ^3*^ZnP.

In **5 c‐GNP** (Figures 8 b, and 9 c,d), SAS3 shows, on one hand, ground state recovery with 1200 ps and, on the other, population of ^3*^ZnP (SAS4) both as charge recombination processes. The Fc^.+^‐ZnP‐GNP^.−^ could not be observed for the **5 c‐GNP**. This is probably due to the relatively low population of Fc^.+^‐ZnP^.−^‐GNP state stemming from the large distance between the Fc and ZnP. Subsequently Fc^.+^‐ZnP‐GNP^.−^, which is the product of charge‐shift gets hardly populated. It is the concomitant formation of the ^3*^ZnP, which dominates the differential absorption spectra across the entire spectral range on longer timescales.

## Conclusion

**Figure 10:**
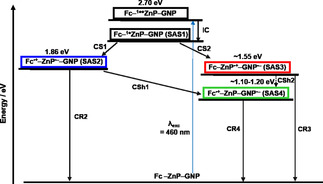
The kinetic model applied to deconvolute the excited state species in the conjugates. ZnP/ZnP^•+^=0.90 V, ZnP/ZnP^•−^=1.32 V, Fc/Fc^•+^=0.54 V, and GNP/GNP^•−^=(−0.5)–(−0.8 V) (IC: internal‐conversion, CS: charge‐separation, CSh: charge‐shift, CR: charge‐recombination). The dashed arrow indicates that the process could not be deconvoluted.

We present the synthesis, characterization, and investigation of novel GNP‐based electron donor‐acceptor conjugates. For the first time, electron‐donating ferrocenes (Fc) were combined with light‐harvesting/electron‐donating (metallo)porphyrins (ZnP) and electron‐accepting GNPs: Fc‐ZnP‐GNP. Different analytical and spectroscopic techniques have been applied to demonstrate the successful GNP functionalization: Mass spectrometry, NMR spectroscopy, XPS, and Raman spectroscopy coupled with Raman imaging. The covalent GNP functionalization enabled reproducible and comparative assays regarding the deactivation dynamics in the Fc‐ZnP‐GNPs, in which variable distances between the GNPs, ZnPs, and Fcs have been guaranteed by employing oligo‐*p*‐phenyleneethynylenes of variable lengths. Of great importance is the fact that cascades of charge‐transfer events (Figure 10) generate long‐lived charge‐separated states featuring a GNP‐delocalized electron and a one‐electron oxidized ferrocenium. Key to this separation of charges is the simultaneous formation of two short‐lived charge‐separated states as intermediates, that is, Fc^.+^‐ZnP^.−^‐GNP and Fc‐ZnP^.+^‐GNP^.−^.

## Conflict of interest

The authors declare no conflict of interest.

## Supporting information

As a service to our authors and readers, this journal provides supporting information supplied by the authors. Such materials are peer reviewed and may be re‐organized for online delivery, but are not copy‐edited or typeset. Technical support issues arising from supporting information (other than missing files) should be addressed to the authors.

Supplementary
