# Supplementary material for: Designing Cascades of Electron Transfer Processes in Multicomponent Graphene Conjugates
Source: Angew Chem Int Ed Engl. 2020 Oct 15;59(52):23706–15. doi: 10.1002/anie.202008820 (PMC7756474; doi:10.1002/anie.202008820)
Supplement: Supplementary file 1 — Supplementary [file ANIE-59-23706-s001.pdf]

Angewandte Chemie
Eine Zeitschrift der Gesellschaft Deutscher Chemiker

Supporting Information

Designing Cascades of Electron Transfer Processes in Multicomponent Graphene Conjugates

Francesca Limosani+, Ramandeep Kaur+, Antonino Cataldo, Stefano Bellucci, Federico Micciulla, Robertino Zanoni, Angelo Lembo, Bingzhe Wang, Roberto Pizzoferrato, Dirk M. Guldi, and Pietro Tagliatesta**

anie_202008820_sm_miscellaneous_information.pdf

Supporting Information

Table of Contents

Experimental Section 4
General method 4
Chemicals 5
Compound 5a. 5
Compound 5a-GNP 5
Compound 5b-GNP 5
Compound 4c 5
Compound 5c 5
Compound 5c-GNP 6
Compound 5-GNP. 6
Chemical Structures 7
ZnTPP, HZnPa, and HZnPb 7
β-ZnP-ref 7
5-GNP 8
Mass Spectrometry and ^{1}H-NMR Spectroscopy 9
HRMS-ESI spectrum of 5a 9
^{1}H NMR in $CDCl_3$ of 5a 10
HRMS-ESI spectrum of 4c 11
^{1}H NMR in C_6D_6 of 4c 12
HRMS-ESI spectrum of 5c 13
^{1}H NMR in C_6D_6 of 5c 14
X-Ray Photoelectron Spectroscopy 15
5a-GNP 15
5c-GNP 16
5-GNP 17
Raman Spectroscopy and Raman Imaging 18
5c -GNP 18
5b-GNP 19
5a-GNP 20
Atomic force microscopy 21
Graphene-nanoplate- GNP 21
5-GNP 22
5a-GNP 23
5b-GNP 24

Photophysical Studies 25
Steady-State Spectroscopy 25
Electrochemical Studies 33
Time-resolved absorption spectroscopy 35

Experimental Section

General method

^{1}H NMR spectra were measured on a Bruker AM-400 and Bruker 300 instruments. The chemical shifts were referenced to the residual peaks of the deuterated solvents: chloroform (7.26 ppm) and benzene (7.15 ppm).
ESI-HRMS were performed with Orbitrap Q-Exactive (Thermo Scientific) in positive ion full scan mode by adopting the following parameters values: spray voltage between 5-6 kV depending on the compound, S-lens RF level = 100, sheath gas level = 50, auxiliary gas level = 10, capillary temperature = 320 °C, auxiliary gas heather = 300 °C, AGC target = 3x10e6, Max inj. Time = 120 ms, resolution power = 100000. Calculation of exact mass or each compound was performed by using Xcalibur software (Thermo Scientific) with an accuracy ≤ 5ppm.
Porphyrin samples (≃ 0.5 mg) were dissolved in toluene, the obtained solution was further diluted 1/10 and 20 □l of this final solution was then added to a 400 □l of ACN:H_2O=4:1 mixture (in case of compound 4c the final ACN/H_2O ratio contained 0.1% g/vol of formic acid).
Raman spectra were obtaining by using a confocal Raman microscope Invia Renishaw, endowed with a 633 nm laser, a RenCam CCD detector, 1024x256 pixels (200-1060 nm), an encoded xyz stage (replacement precision: 100nm), using an 1800 l/mm grating. About mapping two measurements for each point were accumulated using a sampling step of 100 nm. After obtaining the mapping, the spectra were analyzed using the Wire® 4.0 software to elaborate the curve fit. We choose to analyze the peak area data to decrease the error about the estimation of porphyrin localization.
XPS measurements were obtained from a modified Omicron NanoTechnology MXPS system. The spectra were excited by achromatic Mg Kα and Al Kα photons (hv = 1253.6 and 1486.6 eV, respectively), generated operating the anode at 14-15 kV, 10-20 mA. All the samples were mounted on metal tips as thin layers of pressed powders. Experimental spectra were theoretically reconstructed by fitting the peaks to symmetric pseudo-Voigt functions (linear combination of gaussian and lorentzian peaks) and the background to a Shirley or a linear function. XPS atomic ratios (±10% associated error) between relevant core lines were estimated from experimentally determined area ratios corrected for the corresponding theoretical cross sections and for a square root dependence of the photoelectron's kinetic energies. All binding energies were referenced to the lowest lying C 1s peak component, taken at 285.0 eV, which was assumed to be related to the aromatic C atoms.
SEM measurements were carried out by a scanning electron microscope TESCAN VegaII at 10 KV of voltage acceleration with a working distance of 9.788mm. It endowed with Bruker microanalysis and QUANTAX 400 software of analysis, as well as a detector STEM for the acquisition of images in brigthfield and in darkfield.
Atomic force microscopy measurements were carried out with a JPK Nanowizard 4 Nanoscience microscope. The ACTA cantilever from Applied Nanostructures APPNANO with a resonance frequency of 300 kHz and a tip radius below 10 nm was used. The samples were drop casted onto silica wafers.
Cyclic voltammetry (CV) experiments were performed with an EmStat potentiostat, using a platinum wire as counter electrode, Ag/AgCl as reference electrode and a glassy carbon (GCE) as working electrode. All the measurements were carried out at room temperature in a 0.1 M solution of tetra-n-butylammonium hexafluorophosphate ($TBAPF_6$) in acetonitrile, with ferrocene as internal standard at 100 mV/s scan rate. Prior to each voltammetric measurement, the solution was degassed by bubbling with argon for about 20 mins.
For each GNP-conjugate a dispersion was prepared by dissolving 1 mg in 1 mL of anhydrous *N,N*-dimethylformamide. The solutions were bath-sonicated for 30 min at 59 kHz. In the last step, 12 µL of the solutions were drop-casted onto the GCE working electrode and further drying. For the steady -satate and time resolved spectroscopic measurements the dispersions were prepared by sonicating the conjugates in THF for 30-40 mins at 30 kHz and 50 % power. The supernatant was obtained post centrifugation.
A Perkin Elmer Lambda 2 spectrometer was used in order to collect the UV−Vis spectra at 298 K using a slit width of 2 nm and a scan rate of 480 nm /min. The data were recorded with the help of UV WinLab software.
Steady-state fluorescence studies were conducted using Fluoromax 3 spectrometer (Horiba Scientific).
Time-resolved absorption studies were performed by using the Clark MXR CPA 2101 and CPA2110 Ti: sapphire amplifier (775 nm, 1 kHz, 150 fs pulse width) as the laser source. Ultrafast Systems HELIOS femtosecond transient absorption spectrometer was used to acquire time resolved transient absorption spectra with 150 fs resolution and time delays from 0 to 5500 ps. The probe-visible white light (~400−770 nm)- was generated by focusing a fraction of the fundamental 775 nm output onto a 2 mm sapphire disk. And, for the (near) IR (780−1500 nm), a 1 cm sapphire was used. Non-collinear optical parameter (NOPA, Clark MXR) was used to generate the excitation wavelength at 568 nm; a bandpass filter ± 5 nm was used to exclude the fundamental 775 nm and 387 nm. All measurements were performed in 1 cm quartz cuvettes under argon atmosphere. The data points in the 450–470 nm, and 765–790 nm regimes were removed as they stem from the pump excitation and fundamental excitation wavelength, respectively. In order to deconvolute associated species, the spectral data were subjected to global-target analysis, which is based on an excited-state deactivation kinetic model. The plausible kinetic model which was used to fit the data is based on the information obtained on the energetics of the system from steady-state spectroscopy and electrochemistry. To carry out the analysis we used the free software program GloTarAn which is a graphical interface to the R-package TIMP.
Origin 2018/2019/2020-pro version was used to plot the data obtained using different spectroscopic and analytical methods.

We performed the Tauc plot analysis in order to determine the optical band-gap of the GNP from the absorption characteristics using the following equation:

$$(ahv)^{1/n} = A(hv - E_g)$$

where A is the constant, E_g is the band gap of the material and n is the exponent which depends on the type of transition. For direct transition n = 1/2 and for indirect transition n = 2. Using the above relation and Tauc plots[1], we extracted the band gap values by extrapolating the linear region of hv vs. $(ahv)^{1/n}$ to the energy axis.

Furthermore, we considered the Stern-Volmer analysis[2] for the reported conjugates, using the following equation.

$$\frac{I_0}{I} = \frac{\tau_0}{\tau} = 1 + K_{sv}[Q]$$
$$K_{sv} = k_q \tau_0$$

In the equation above, K_{sv} is the Stern-Volmer constant, $k_q$is the bimolecular rate constant, I_0 and τ_0 stand for the fluorescence intensity and lifetime, respectively, of the fluorophore in absence of the quencher [Q], and I and τ represents the same in presence of the quencher.

This was done, in order to comment on the impact of GNP and Fc as the two quenchers, on the intrinsic fluorescence and singlet excited state lifetime of the ZnP.

Chemicals

Silica gel 60 (70-230 mesh, Sigma Aldrich) was used for column chromatography. High-purity-grade nitrogen gas was purchased from Rivoira. When anhydrous conditions were required the solvents were freshly distilled under nitrogen atmosphere and at ambient pressure, following the literature procedures: toluene was distilled over sodium and THF over $LiAlH_4$. All the reagents and solvents were from Fluka Chem. Co., Aldrich Chem. Co. or Carlo Erba and were used as received.
1 (H_2Br_2) compound was obtained as reported in literature.[47]
3, **4a**, and **5b** compounds were previously reported in literature[34] while **4b** compound was synthetized and reported in literature.[44]
5a-C60 and **β-ZnP-ref** were synthetized and reported in literature.[32]

Compound 5a.

50 mg (0.048 mmol) of **4a**, were dissolved in 55 mL of chloroform and 2 mL of saturated solution of $Zn(AcO)_2$ in methanol was added. Mixture was refluxed and stirred for 3 h. The solvent was removed under vacuum and the crude product was purified by a plug of silica gel eluting with chloroform. The fraction containing the desired product was collected and the solvent was evaporated. The compound was crystallized from dichloromethane/methanol to give compound **5a** (48 mg, 0.045 mmol, 89%).
ESI-HRMS for $C_{73}H_{44}FeN_4OZn$ $[M]^{•+}$ m/z Calcd: 1112.2156 Found: 1112.2131; ^1H NMR (300 MHz; $CDCl_3$): δ (ppm) 10.06 (s, 1H), 9.00- 8.70 (m, 6H), 8.27-8.24 (m, 8H), 8.00 – 7.32 (m, 20H) 4.36 (s, 2H), 4.26 (s, 7H); UV-vis (CH_2Cl_2): λ_{max} (log ε) 435 (5.38), 562 (4.16), 599 (3.88).

Compound 5a-GNP.

24 mg (0.021 mmol) of **5a**, 47 mg of GNP and 44 mg (0.49 mmol) N-methylglycine were refluxed for 72 h in 20 mL of anhydrous toluene, under nitrogen atmosphere. The reaction mixture was washed on gooch filter several times with toluene to remove unreacted porphyrin, many times with water to remove N-methylglycine and finally with acetone to remove water. The final compound **5a-GNP** was dried at 60° C under vacuum for 10 hours.
UV-vis (THF): λ_{max} 445, 575, and 612 nm

Compound 5b-GNP.

20 mg (0.017 mmol) of **5b**, 40 mg of GNP and 30 mg (0.34 mmol) N-methylglycine were refluxed for 72 h in 20 ml of anhydrous toluene under nitrogen atmosphere. The reaction mixture was washed, on a gooch filter, three times with toluene to remove unreacted porphyrin, many times with water to remove N-methylglycine and three times with acetone to remove water. The final compound **5b-GNP** was dried at 60 °C under vacuum for 10 hours.
UV-vis (THF): λ_{max} 445, 465, 575 ,612 nm

Compound 4c

50 mg (0.054 mmol) of **3c**, 42 mg (0.11 mmol) of **c** and 15 mg (0.047 mmol) of $AsPh_3$ were dissolved in 25 mL of anhydrous toluene and 5 mL of anhydrous triethylamine under nitrogen atmosphere. The solution was deaerated using argon bubbling for 30 min., then 6.5 mg (0.0063 mmol) of $Pd_2(dba)_3$ were added. The solution was deaerated for further 20 min., after that the argon inlet was place 1 cm above the solution. The flow rate was increased slightly, and the reaction was left under nitrogen at 40°C for 16 h. The solvent was evaporated, and the crude product was purified by column chromatography on silica gel eluting with toluene. The fraction containing the desired product was collected and the solvent was removed under vacuum to give compound **4c** (35 mg, 0.03 mmol, 53%).
ESI-HRMS for $C_{87}H_{55}FeN_4O$ $[M+H]^+$ m/z Calcd: 1227.3720 Found: 1227.3684; ^1H NMR (400 MHz, C_6D_6, 25°C): δ 9.55 (s, 1H), 9.41 (s, 2H), 8.91-8.65 (m, 5H), 8.16 (s, 4H), 8.05 (s, 5H), 7.62-7.32 (br m,26H), 4.45 (s, 2H), 4.14 (s, 2H), 3.86 (s, 5H), -2.13 (s, 2H); UV-vis (CH_2Cl_2): λ_{max} (log ε) 433 (5.43), 532 (4.4), 573 (4.25), 608 (3.99), 664 (3.73) nm.

Compound 5c

32 mg (0.026 mmol) of **4c** were dissolved in 34 mL of chloroform and 2 mL of saturated solution of $Zn(AcO)_2$ in methanol was added. The mixture was refluxed and stirred for 3 h. The solvent was removed under vacuum and the crude product was purified by a plug of silica gel eluting with chloroform. The fraction containing the desired product was collected and the solvent was evaporated. The compound was crystallized from dichloromethane/methanol to give compound **5c** (30 mg, 0.023 mmol, 88%).

ESI-HRMS for $C_{87}H_{52}FeN_4OZn$ $[M]^{•+}$ m/z Calcd: 1288.2782 Found: 1288.2757; ^1H NMR (400 MHz, C_6D_6): δ (ppm) 9.57 (s, 1H), 9.56 (s, 1H), 9.54 (s, 1H), 8.95-8.88 (m, 4H), 8.84 (s, 2H), 8.32-8.26 (m, 6H), 8.24-8.18 (m, 8H), 7.61-7.45 (m, 12H), 7.41-7.30 (m, 8H), 4.47 (s, 2H), 4.14 (s, 2H), 3.91 (s, 5H); UV-vis (CH_2Cl_2): λ_{max} (log ε) 441 (5.4), 567 (4.43), 606 (4.44).

Compound 5c-GNP.

25 mg (0.019 mmol) of **5c**, 40 mg of **GNP** and 34 mg (0.38 mmol) N-methylglycine were refluxed for 72 h in 25 ml of anhydrous toluene under nitrogen atmosphere. The mixture reaction was washed, on a gooch filter, several times with toluene to remove unreacted porphyrin, many times with water to remove N-methylglycine and finally with acetone to remove water. The final compound **5c-GNP** was dried at 60°C under vacuum for 10 hours.
UV-vis (THF): λ_{max} 447, 465, 575, and 612 nm

Compound 5-GNP.

50 mg (0.06 mmol) of Zinc 2-[(4’-formyl)phenyl]ethynyl-5,10,15,20-tetraphenylporphyrin, 100 mg of **GNP** and 70 mg (0.79 mmol) N-methylglycine were refluxed for 72 h in 35 ml of anhydrous toluene under nitrogen atmosphere. The reaction mixture was washed, on a gooch filter, several times with toluene to remove unreacted porphyrin, many times with water to remove N-methylglycine and finally with acetone to remove water. The final compound **5-GNP** was dried at 60°C under vacuum for 10 hours.
UV-vis (THF): λ_{max} 436, 564, and 600 nm.

Chemical Structures

ZnTPP, HZnPa, and HZnPb

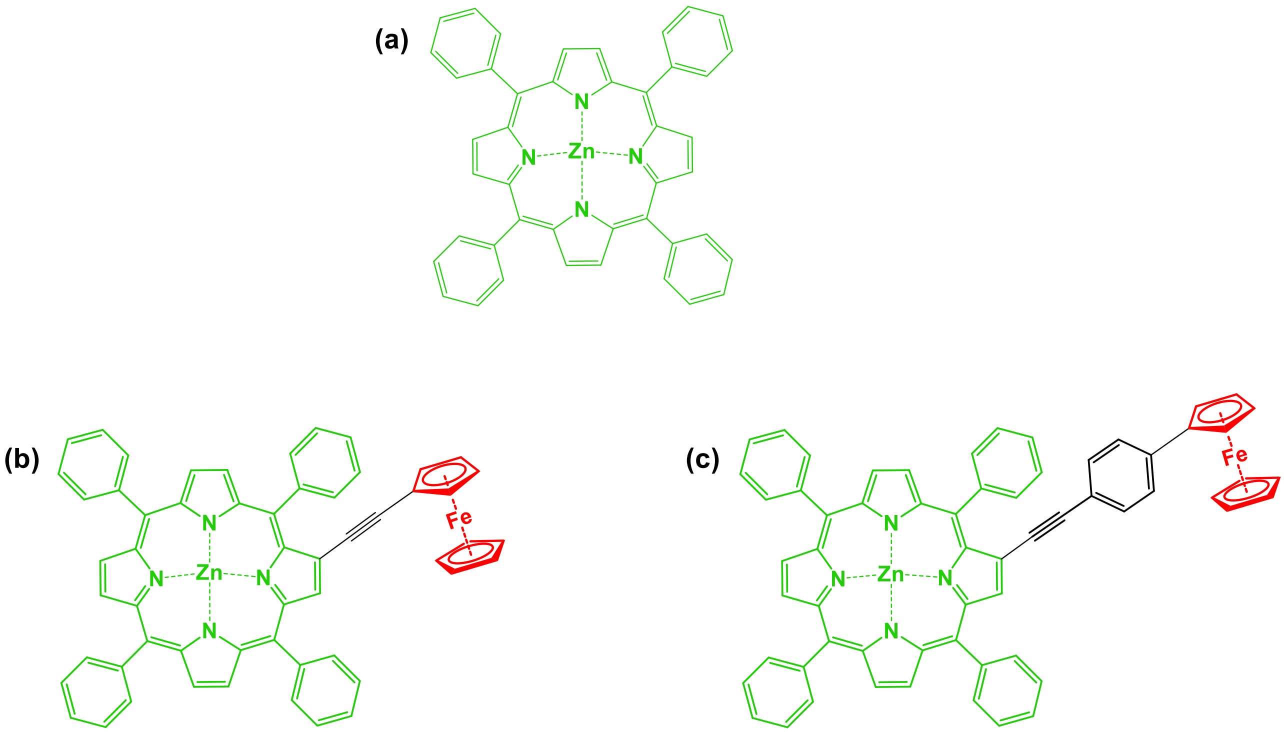

Figure S1. Structures of (a) **ZnTPP**, (b) **HZnPa**, and (c) **HZnPb**
HZnPa and **HZnPb** are used as reference for ZnP-Fc based conjugates.

β-ZnP-ref

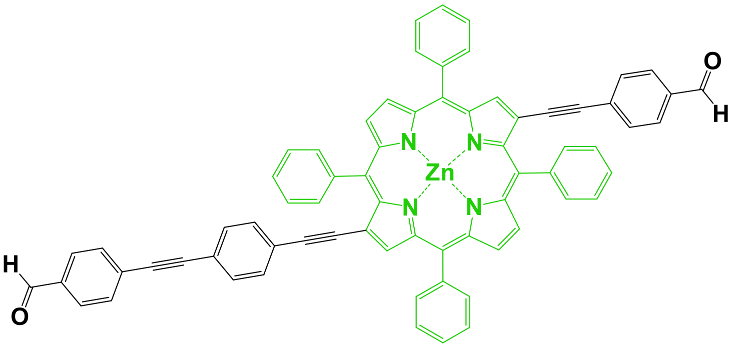

Figure S2. Structure of the β-substituted reference **β-ZnP-ref**.

5-GNP

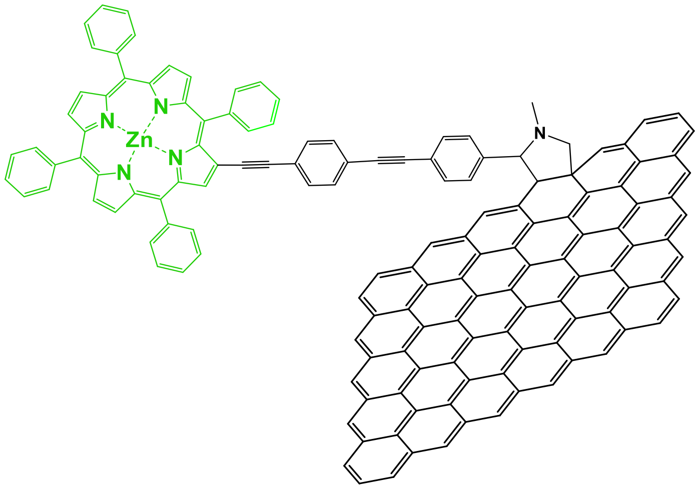

Figure S3. Structure of **5-GNP**.

Mass Spectrometry and ^{1}H-NMR Spectroscopy

HRMS-ESI spectrum of 5a

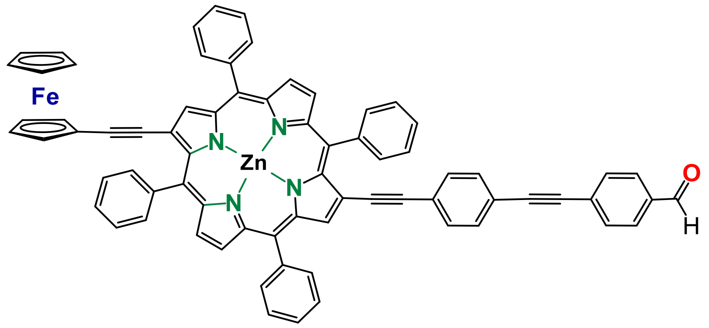

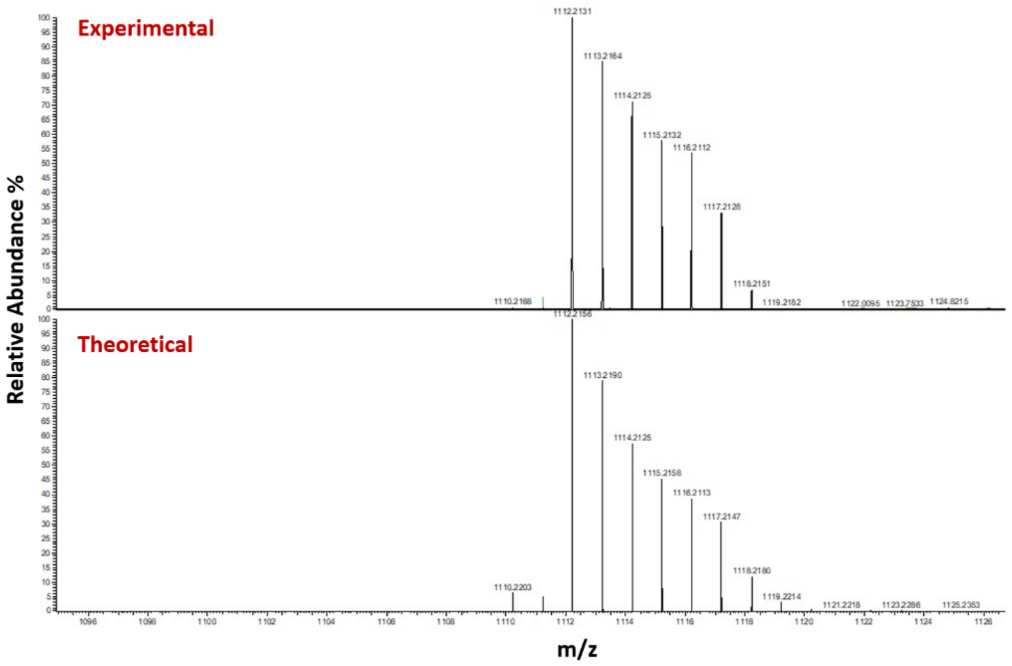

Figure S4. HRMS-ESI spectrum of the molecular peak $[M]^{\bullet+}$ of **5a**.

^{1}H NMR in $CDCl_3$ of 5a

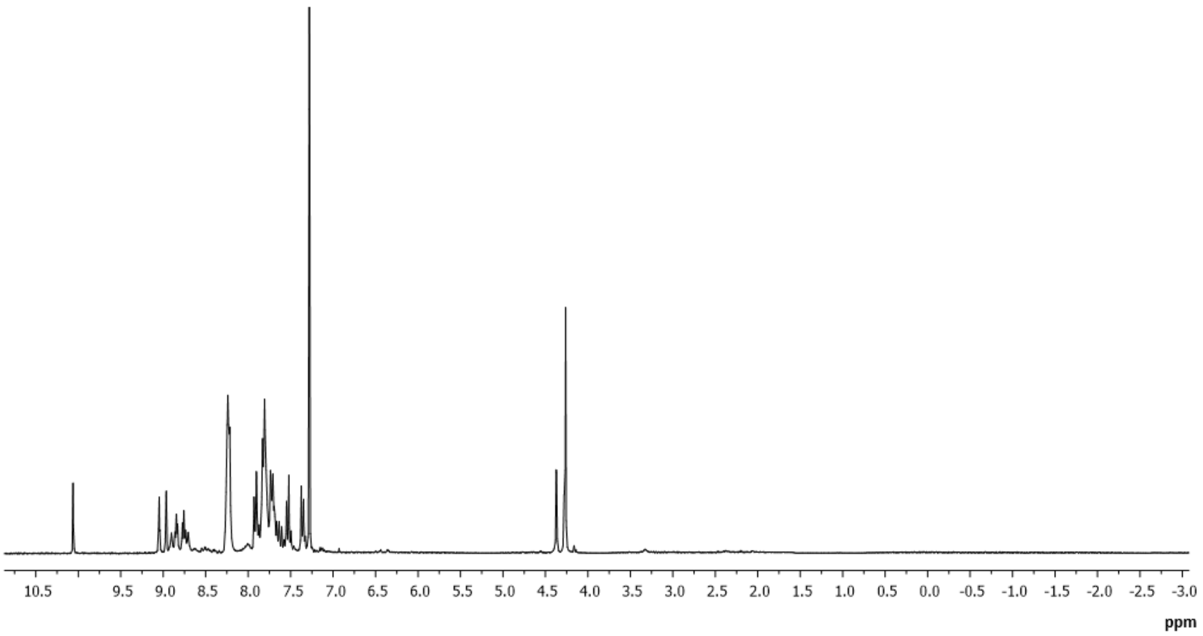

Figure S5. ^{1}H NMR in $CDCl_3$ of **5a** at 300 K and 300 MHz.

HRMS-ESI spectrum of 4c

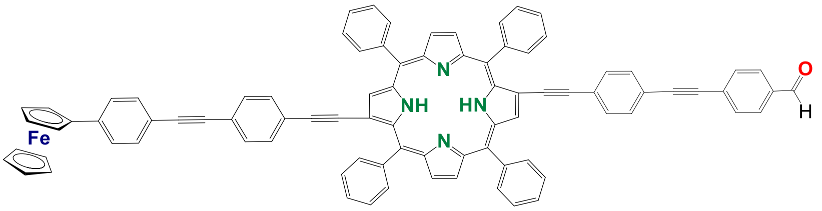

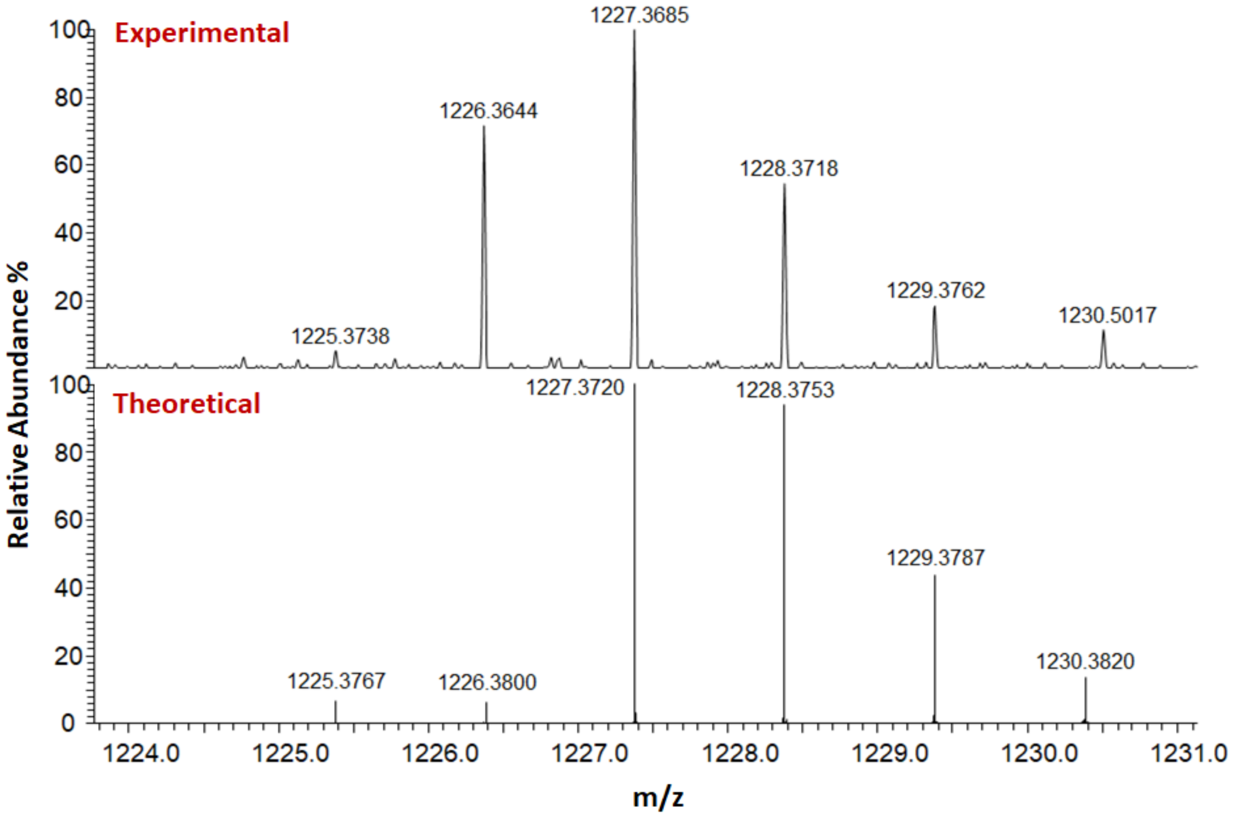

Figure S6. HRMS-ESI spectrum of the molecular peak $[M+H]^{\bullet+}$ of **4c**.

^{1}H NMR in C_6D_6 of 4c

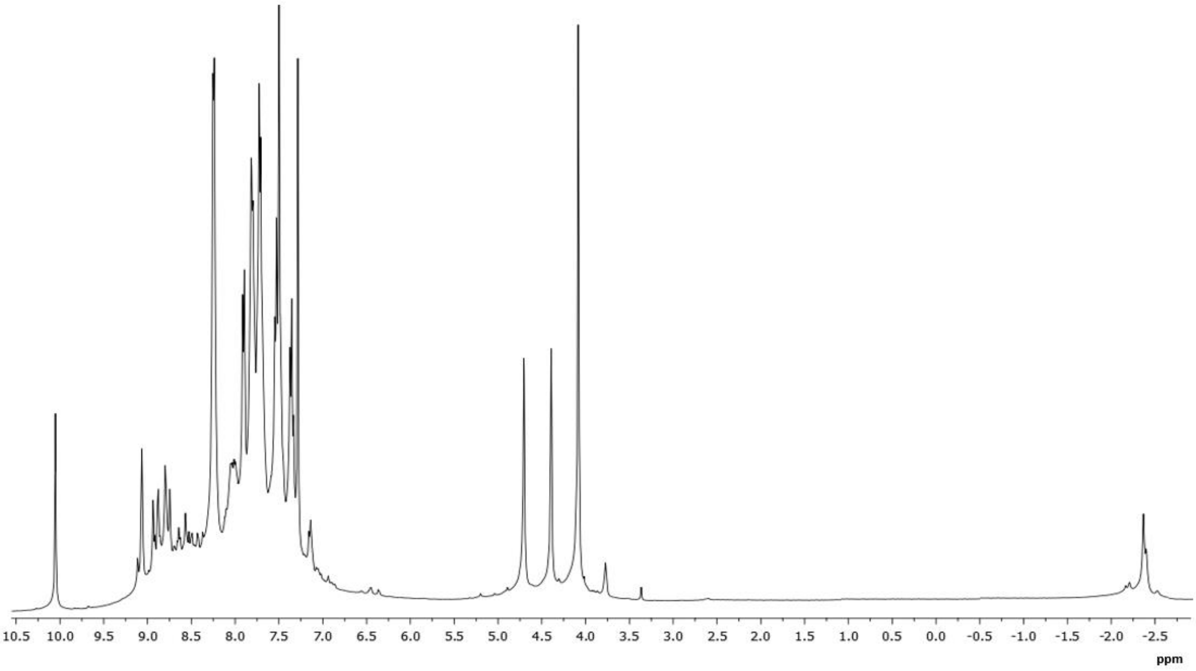

Figure S7. ^{1}H NMR in C_6D_6 of **4c** at 300 K and 400 MHz.

HRMS-ESI spectrum of 5c

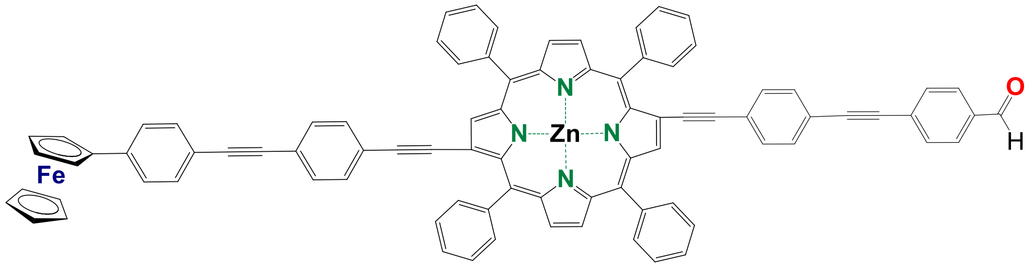

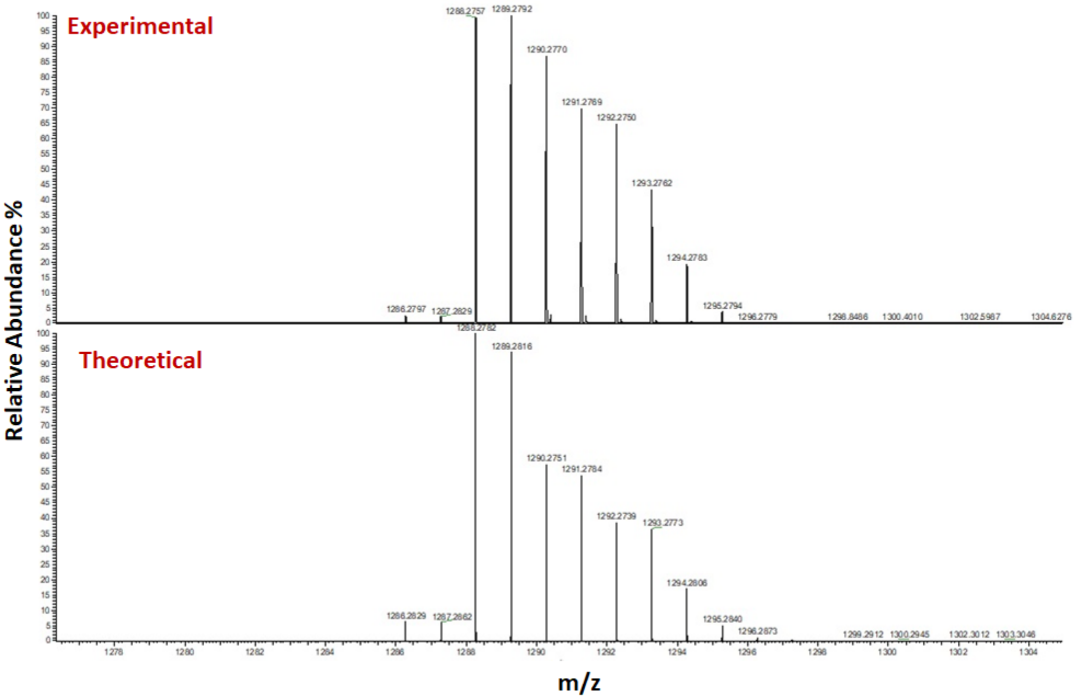

Figure S8. HRMS-ESI spectrum of the molecular peak [M]$^{\bullet+}$ of **5c**.

^{1}H NMR in C_6D_6 of 5c

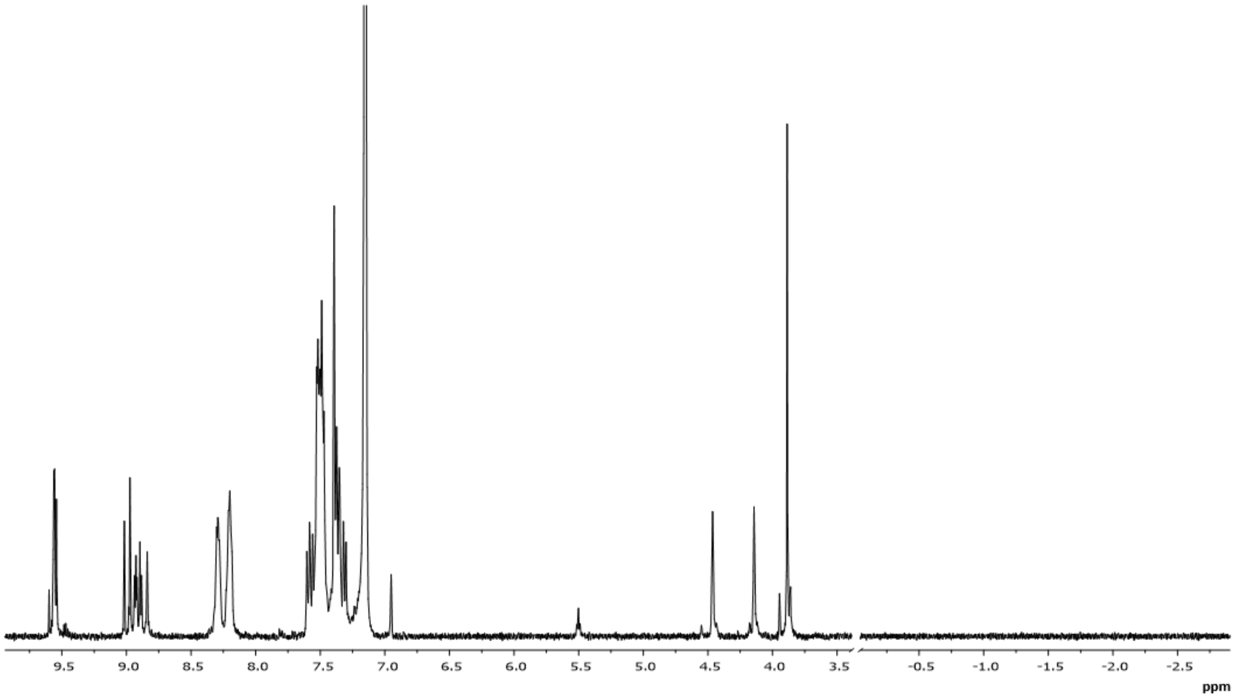

Figure S9. ^{1}H NMR in C_6D_6 of **5c** at 300 K and 400 MHz.

X-Ray Photoelectron Spectroscopy

5a-GNP

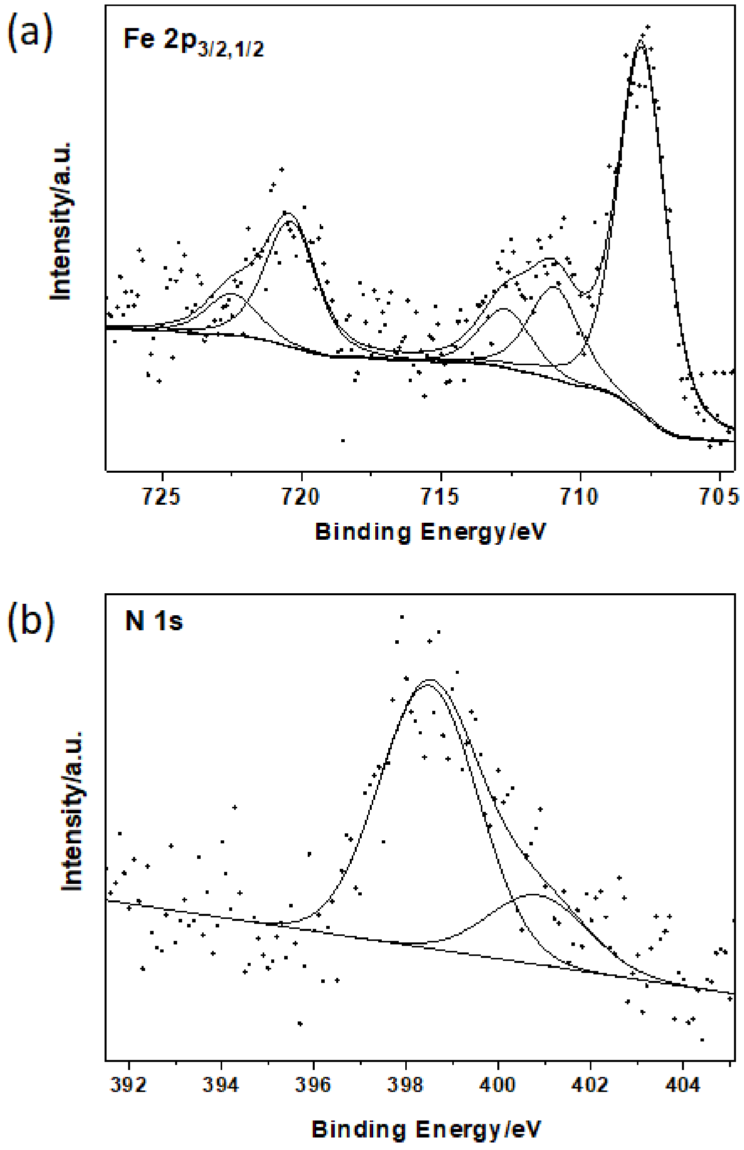

Figure S10. (a) Fe $2p_{3/2,1/2}$ and (b) N 1s photoemission regions of **5a-GNP**. Experimental curve (dots) and peak-fit components (lines) are shown together.

5c-GNP

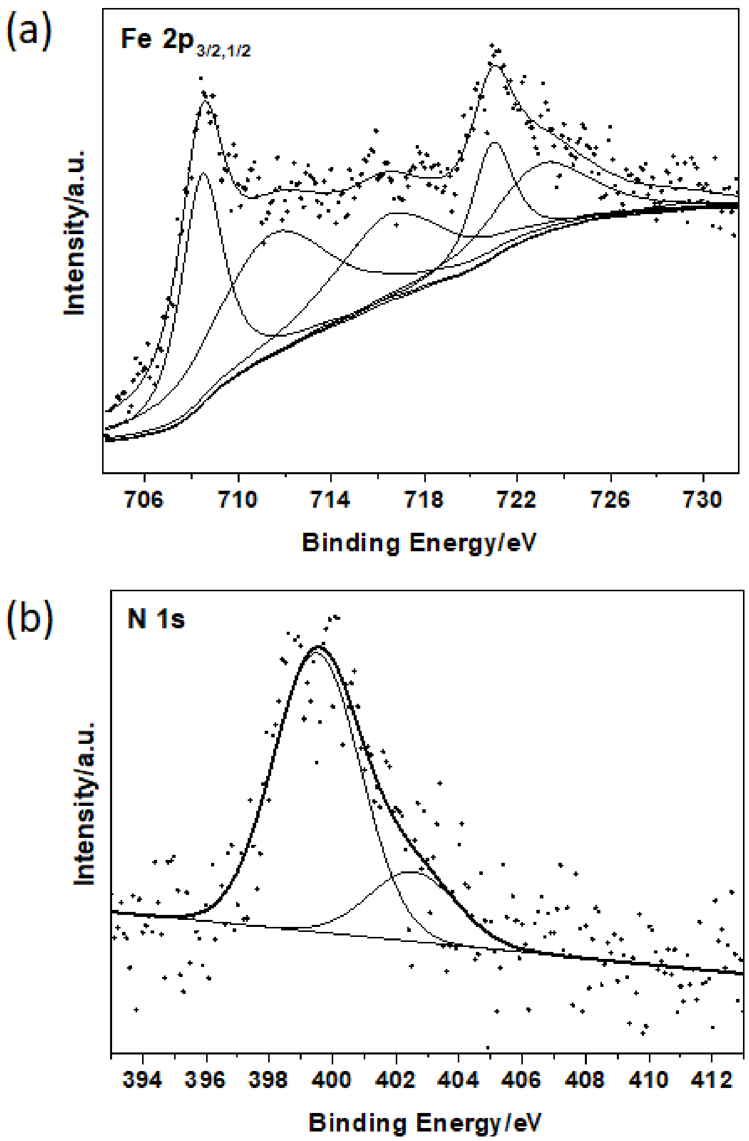

Figure S11. (a) Fe $2p_{3/2,1/2}$ and (b) N 1s photoemission regions of **5c-GNP**. Experimental curve (dots) and peak-fit components (lines) are shown together.

5-GNP

In Figures S12a and S12b the N 1s and Zn $2p_{3/2,1/2}$ photoemission regions for **5-GNP** are reported (Fe is not present in the compound).

The two N 1s distinct components shown in Figure S12a confirm the functionalization of the Zinc 2-[(4'-formyl)phenyl]ethynyl-5,10,15,20-tetraphenylporphyrin on graphene surface. The first peak was attributed to porphyrin nitrogens (399.4 eV) and the second to substituted amine group (402.4 eV) with a 4.0 atomic ratio of the first to the second component. In Figure S12b the Zn $2p_{3/2,1/2}$ component shows a peak at 1022.4 eV, confirming the insertion of the zinc in the macrocycle.

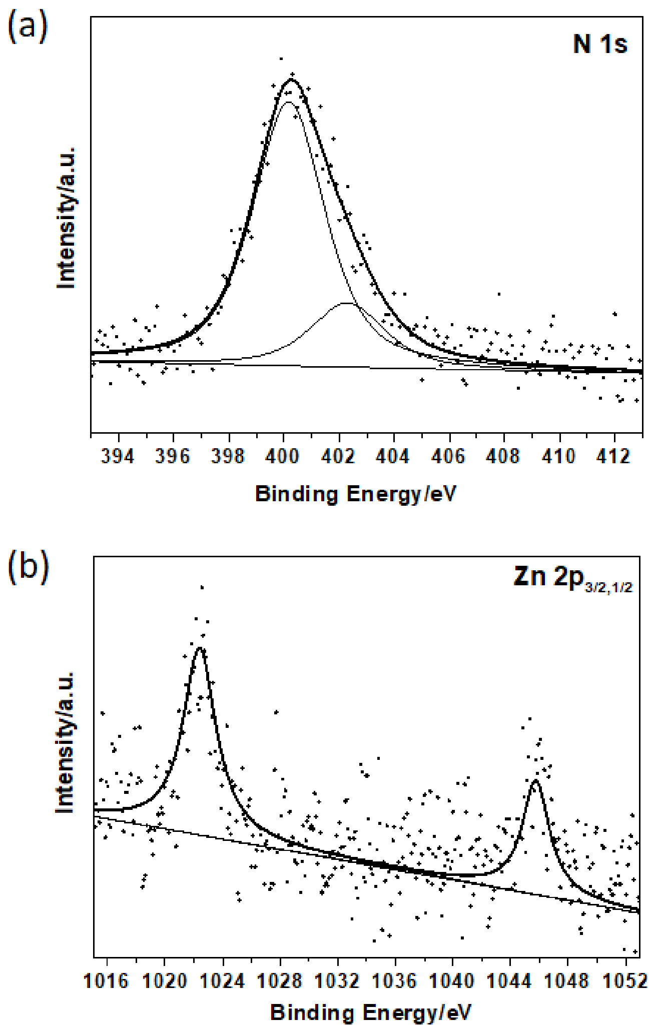

Figure S12. (a) N 1s and (b) Zn $2p_{3/2,1/2}$ photoemission regions of **5-GNP**. Experimental curve (dots) and peak-fit components (lines) are shown together.

Raman Spectroscopy and Raman Imaging

5c-GNP

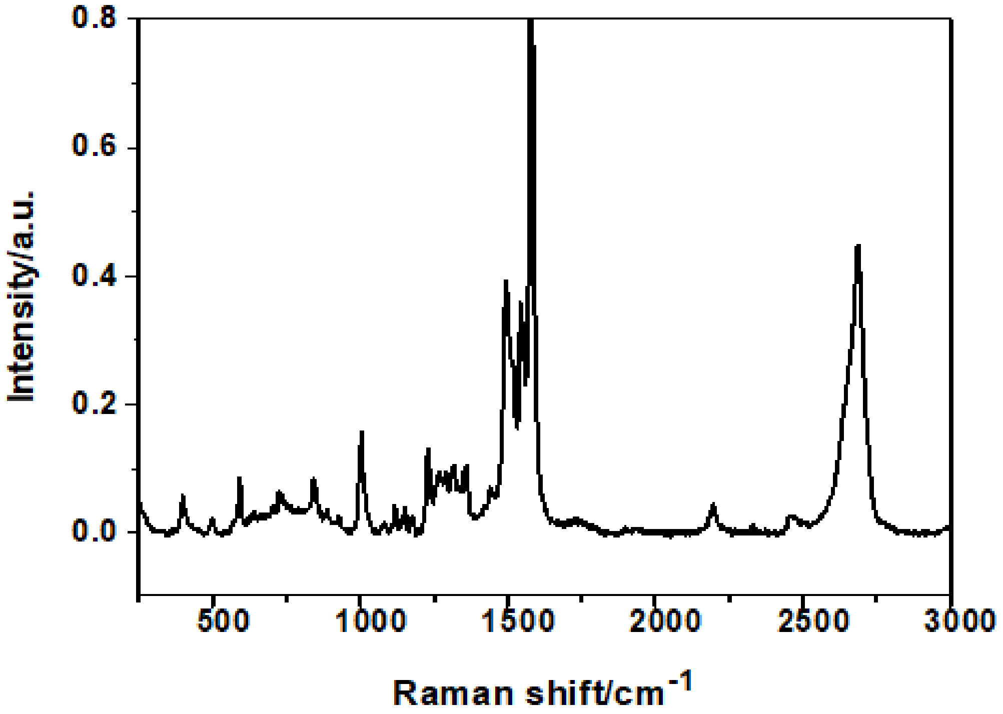

Figure S13. Raman spectrum using a 600 l/mm grating in the spectral range 200 - 3000 cm^{-1} of **5c-GNP**.

5b-GNP

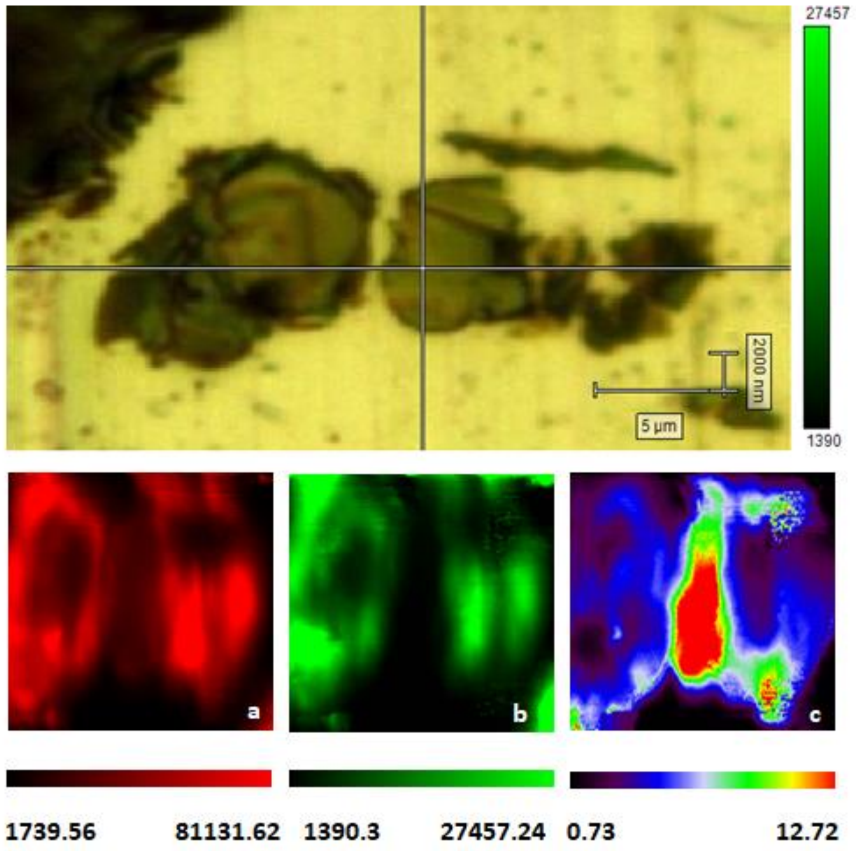

Figure S14. Raman imaging of **5b-GNP**: (a) Peak Area G-band at 1578 cm^{-1}, (b) Peak Area of **5b** at 1498 cm^{-1}, (c) Peak Area G-band/Peak Area of **5b**. Sampling step: 100nm.

5a-GNP

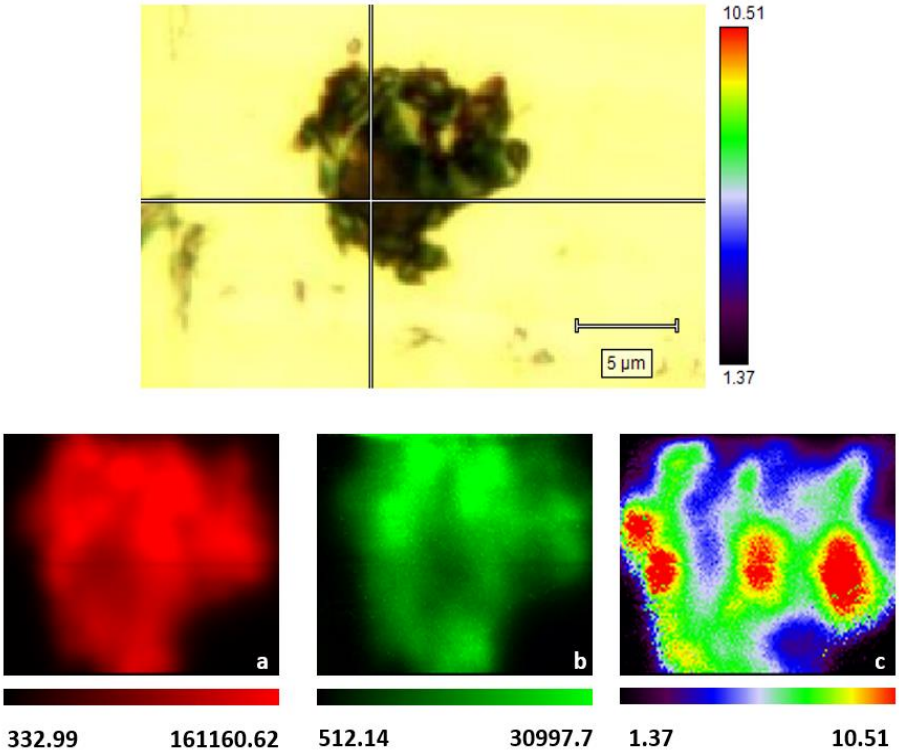

Figure S15. Raman imaging of **5a-GNP**: (a) Peak Area G-band at 1578 cm^{-1}, (b) Peak Area of **5a** at 1498 cm^{-1}, (c) Peak Area G-band/Peak Area of **5a**. Sampling step: 100nm.

Atomic force microscopy

Graphene-nanoplate- GNP

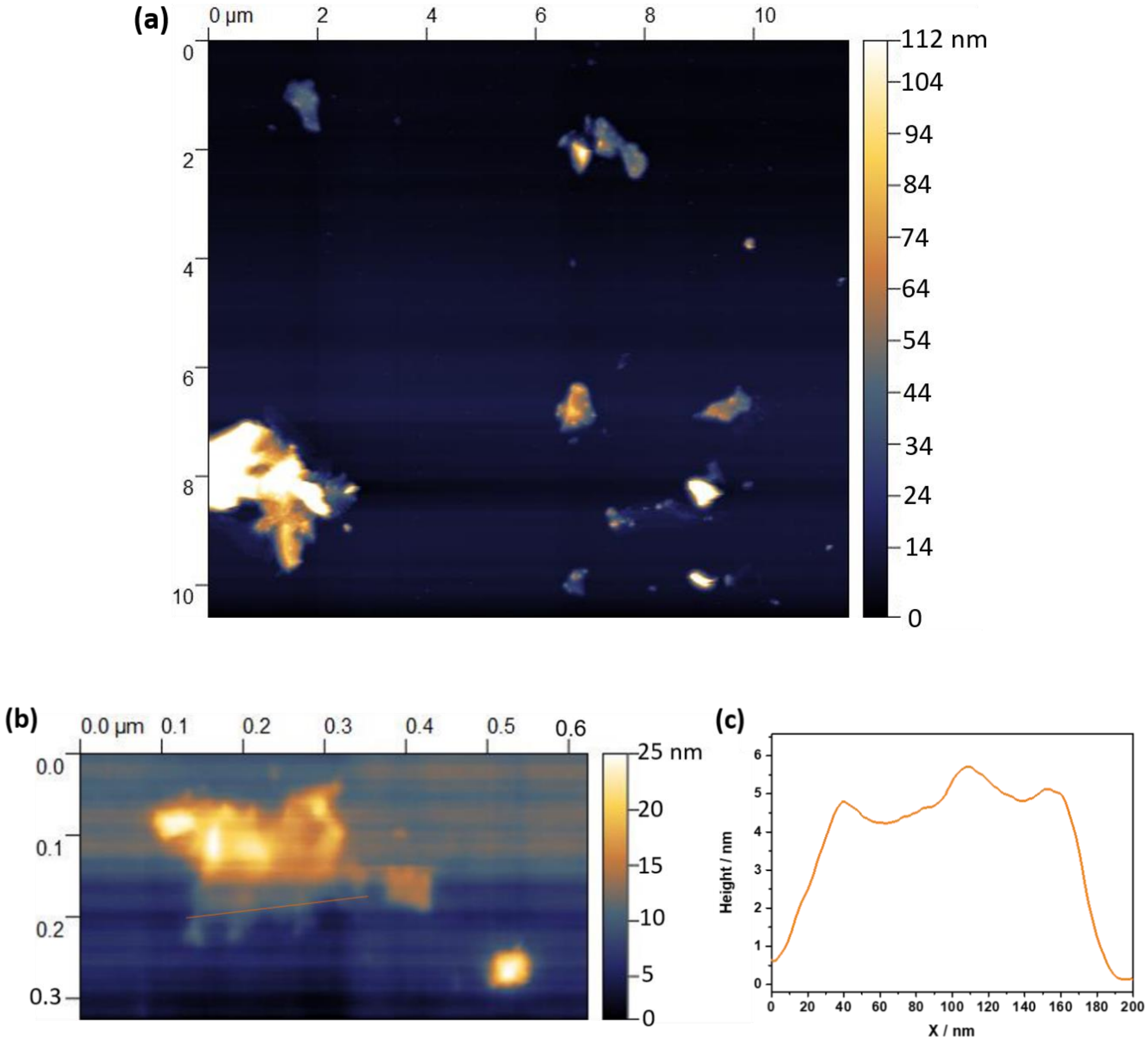

Figure S16. (a) and (b) AFM images and zoom in AFM images, respectively, of **GNP** dispersed in THF and drop casted onto Si/SiO_2 wafer. (c) Exemplary height profile.

5-GNP

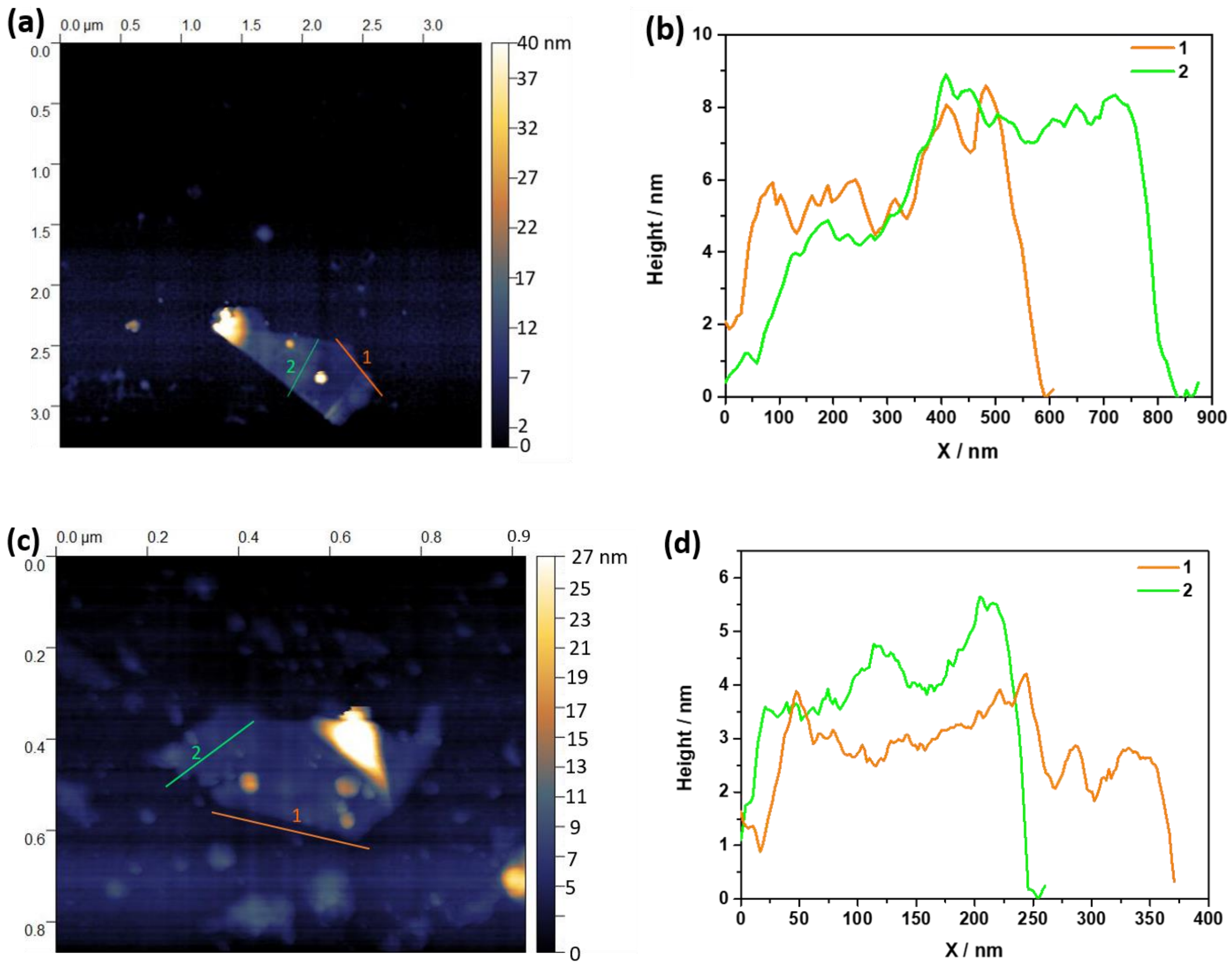

Figure S17. (a) and (c) AFM images of **5-GNP** dispersed in THF and drop casted onto Si/SiO_2 wafer. (b) and (d) Exemplary height profiles.

5a-GNP

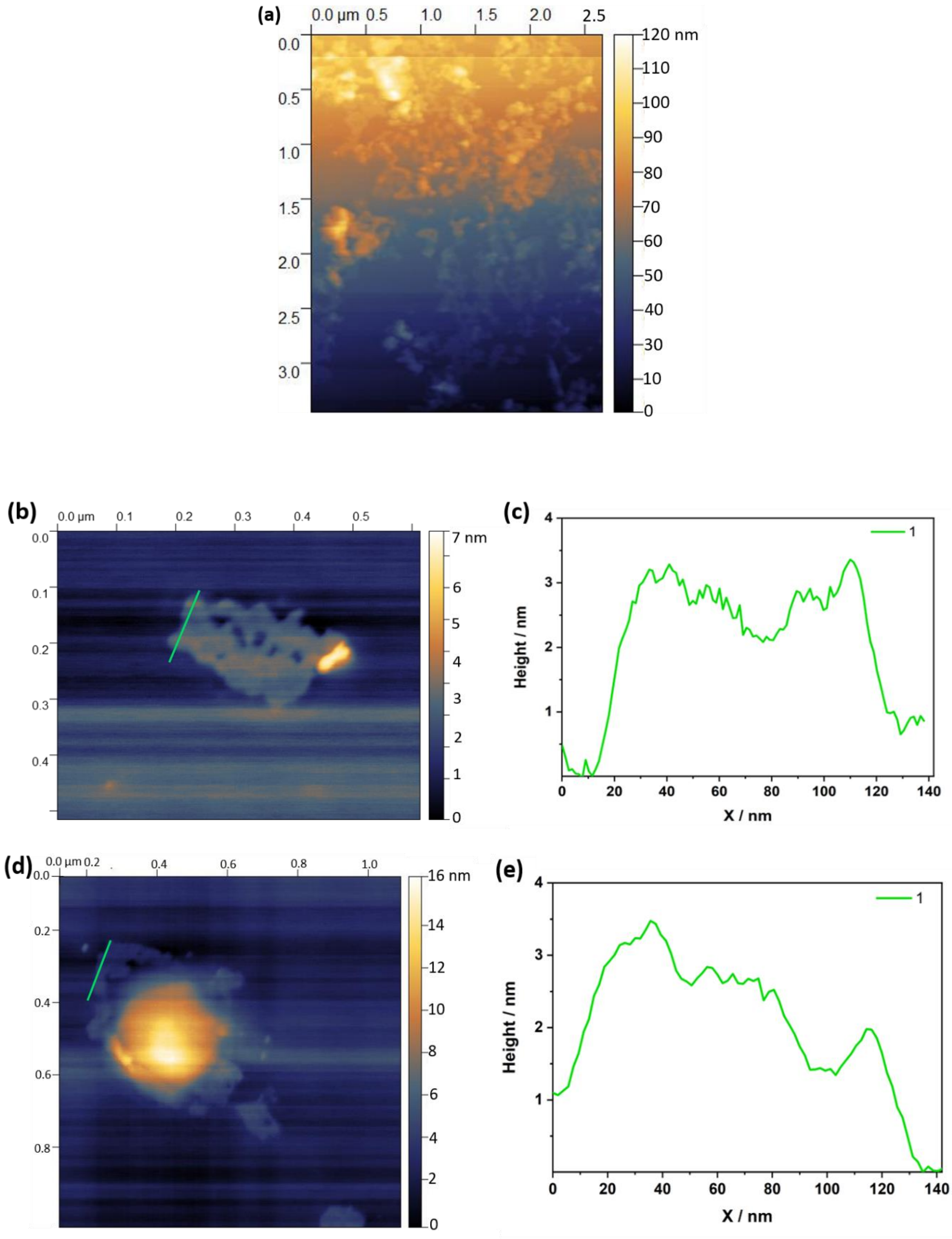

Figure S18. (a), (b), and (d) AFM images of **5a-GNP** dispersed in THF and drop casted onto Si/SiO_2 wafer. (c) and (e) Exemplary height profiles.

5b-GNP

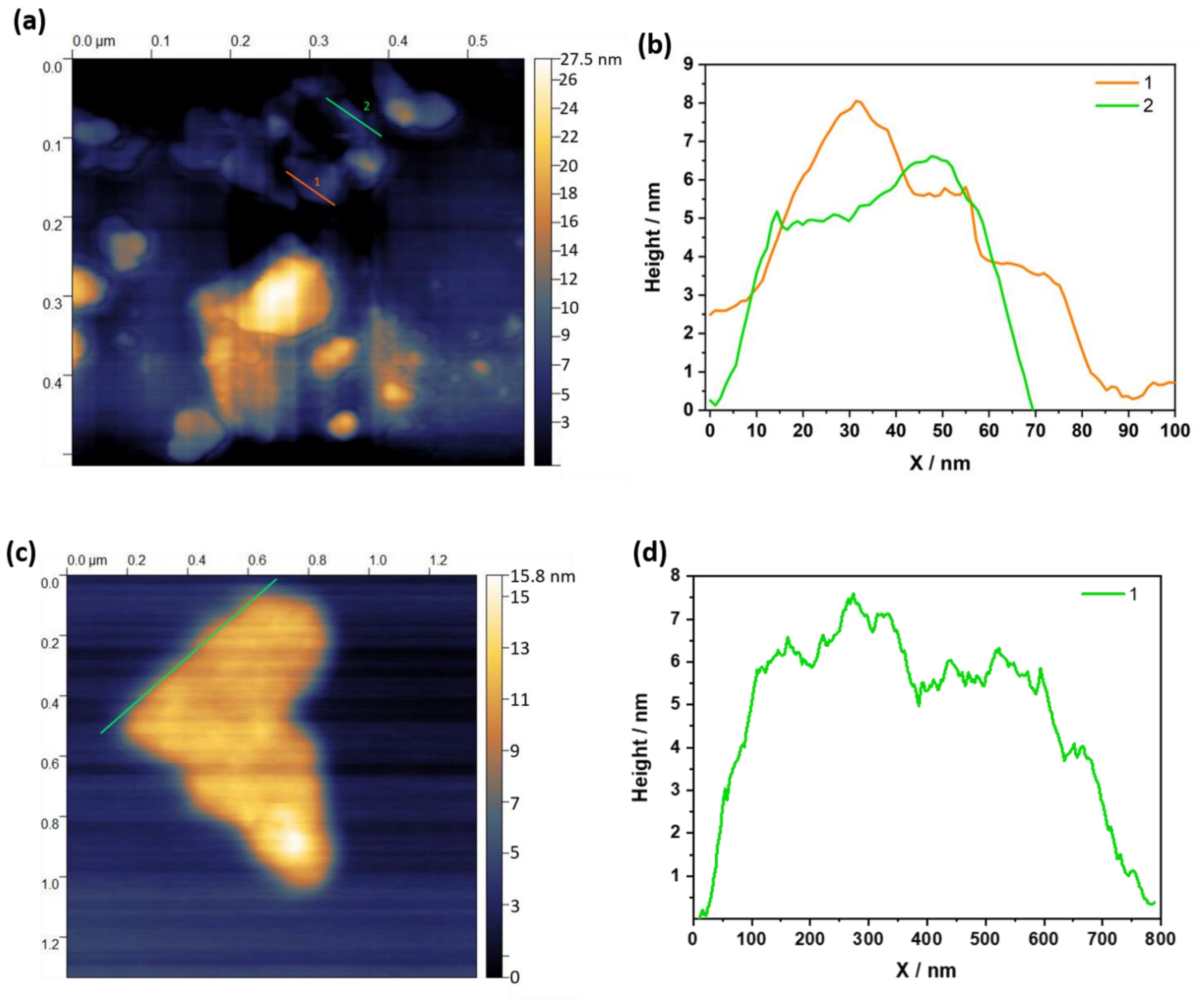

Figure S19. (a) and (c) AFM images of **5b-GNP** dispersed in THF and drop casted onto Si/SiO_2 wafer. (b) and (d) Exemplary height profiles.

Photophysical Studies

Steady-State Spectroscopy

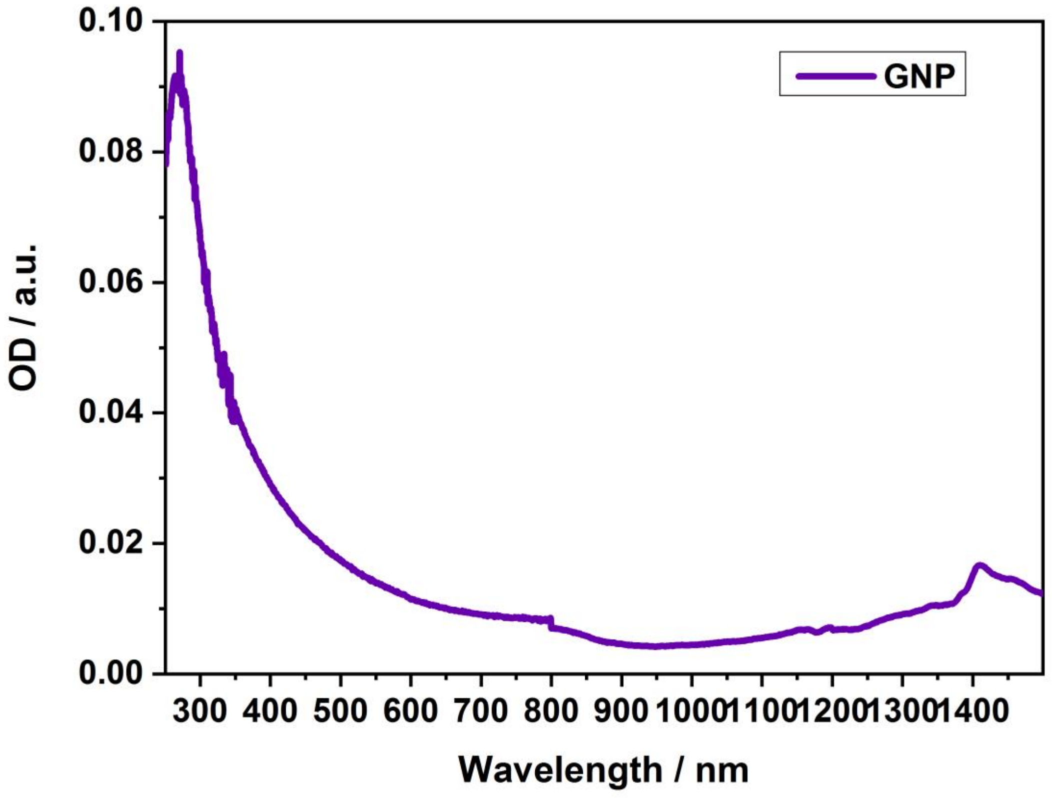

Figure S20. Absorption spectrum of **GNP** (purple) recorded in THF under ambient conditions.

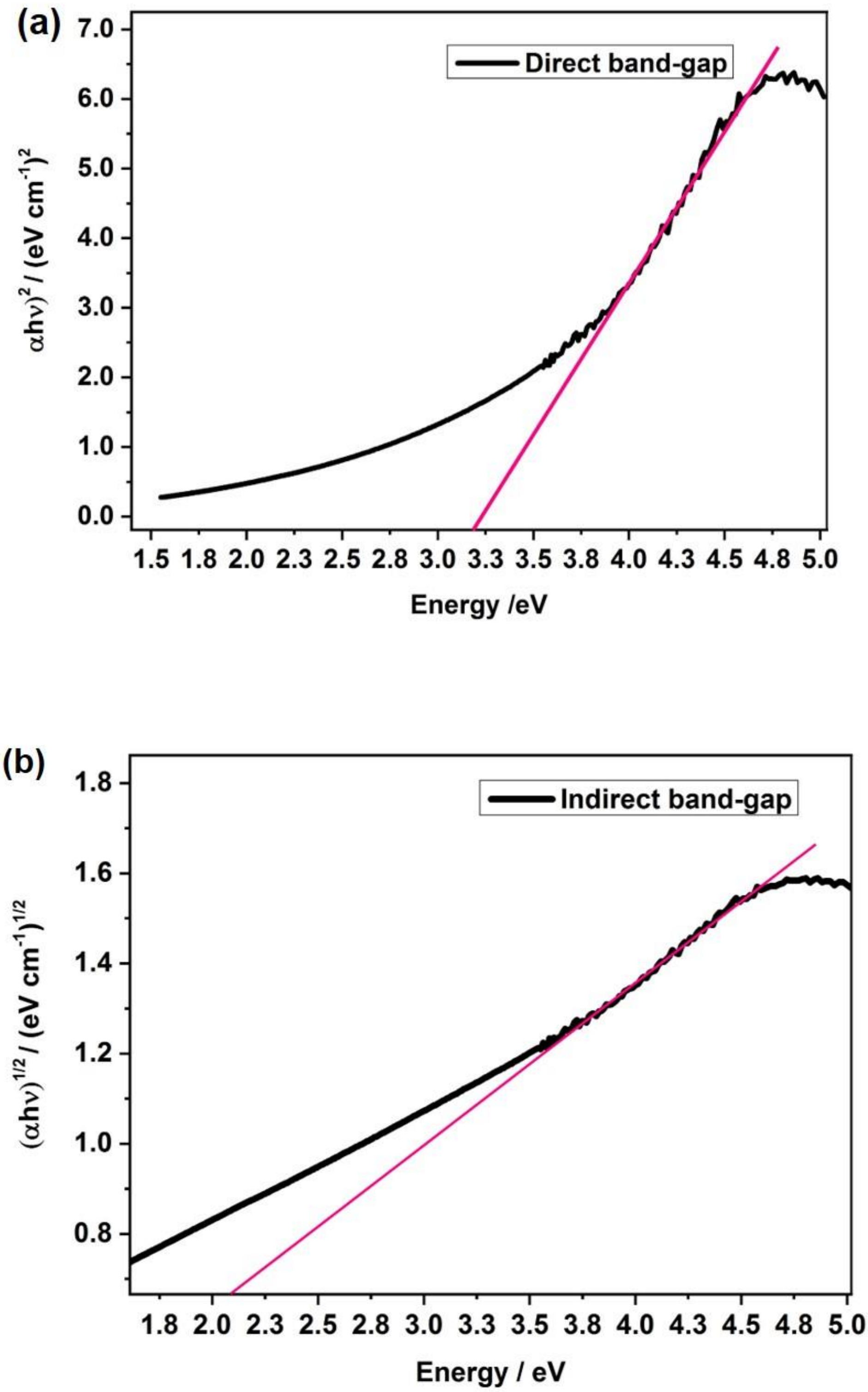

Figure S21. TAUC plots of **GNP** (a) for direct band gap – 3.21 eV and (b) for indirect band gap – 2.10 eV, calculated using the absorption characteristics recorded in THF under ambient conditions.

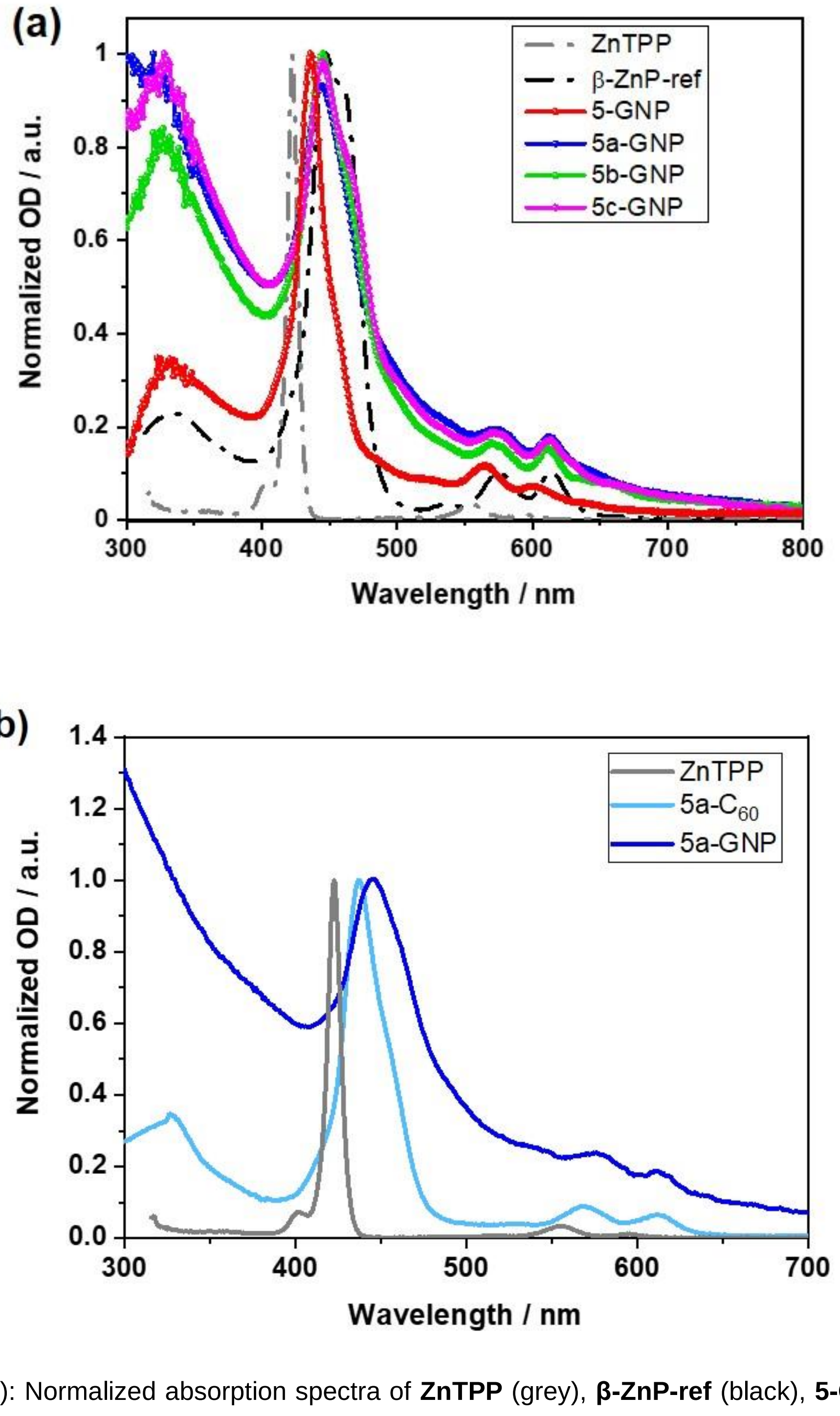

Figure S22. (a): Normalized absorption spectra of **ZnTPP** (grey), **β-ZnP-ref** (black), **5-GNP** (red), **5a-GNP** (blue), **5b-GNP** (green), and **5c-GNP** (magenta), (b): of **ZnTPP** (grey), **5a-C_{60}** (light blue), and **5a-GNP** (blue) recorded in THF under ambient conditions.

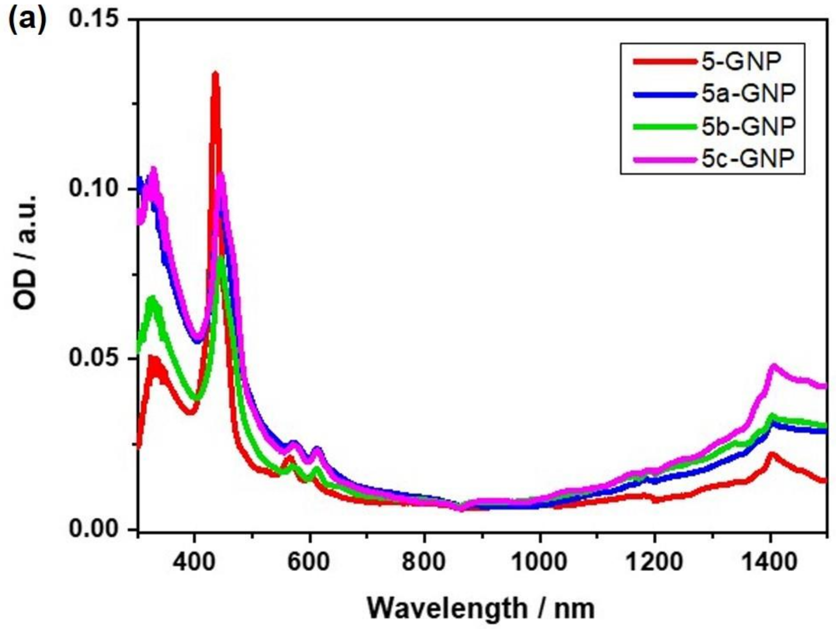

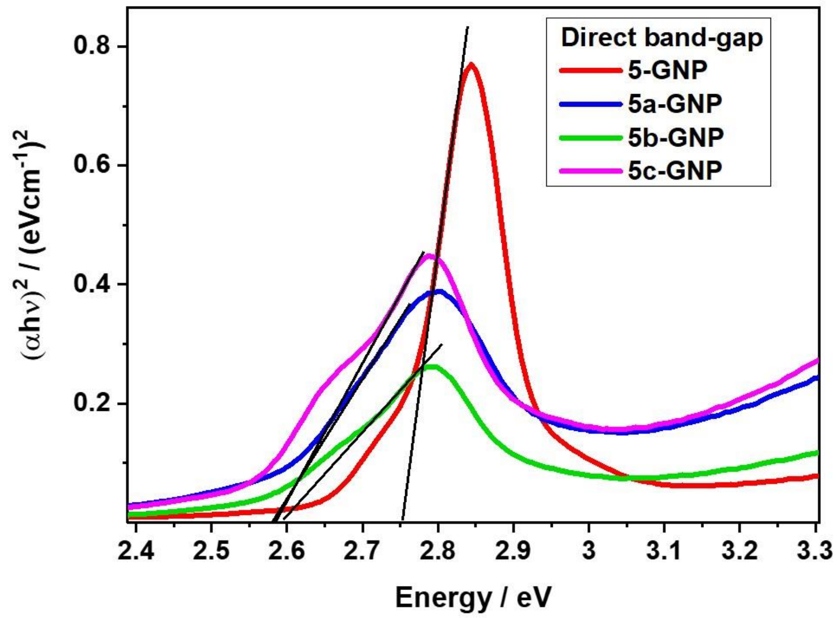

Figure S23. (a): Absorption spectra of **5-GNP** (red), **5a-GNP** (blue), **5b-GNP** (green), and **5c-GNP** (magenta) recorded under ambient conditions in THF. (b): TAUC plots of **5-GNP** (red), **5a-GNP** (blue), **5b-GNP** (green), and **5c-GNP** (magenta) for direct band gap.

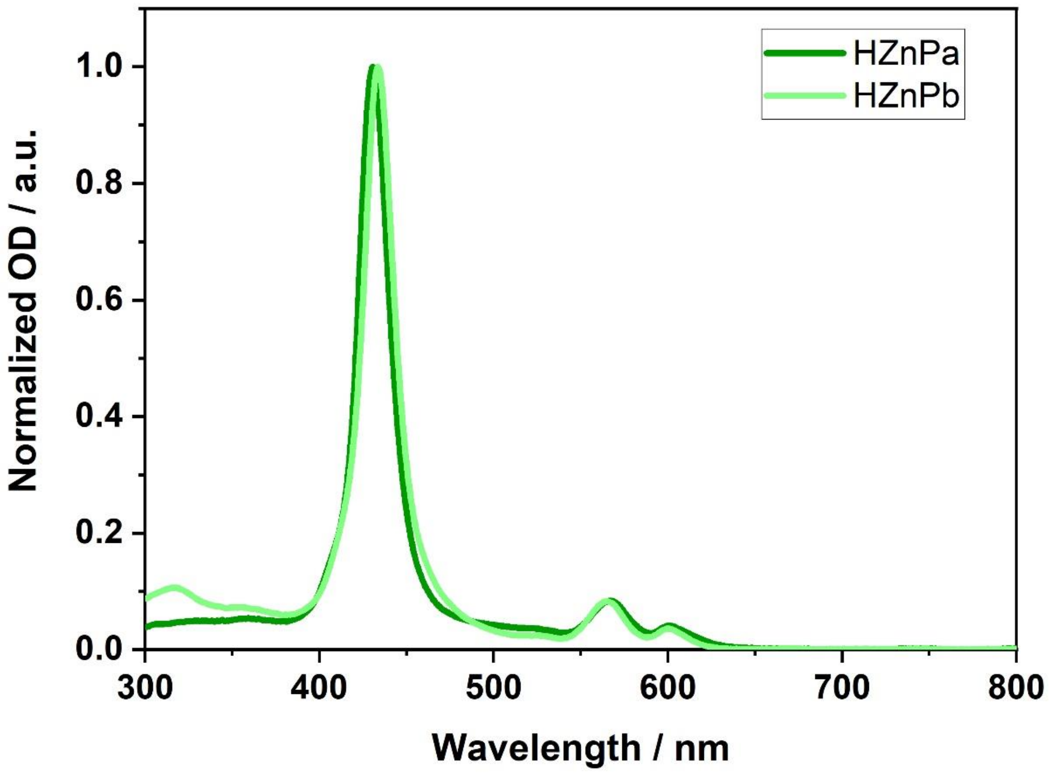

Figure S24. Absorption spectra of **HZnPa** (dark green) and **HZnPb** (light green) recorded in THF under ambient conditions.

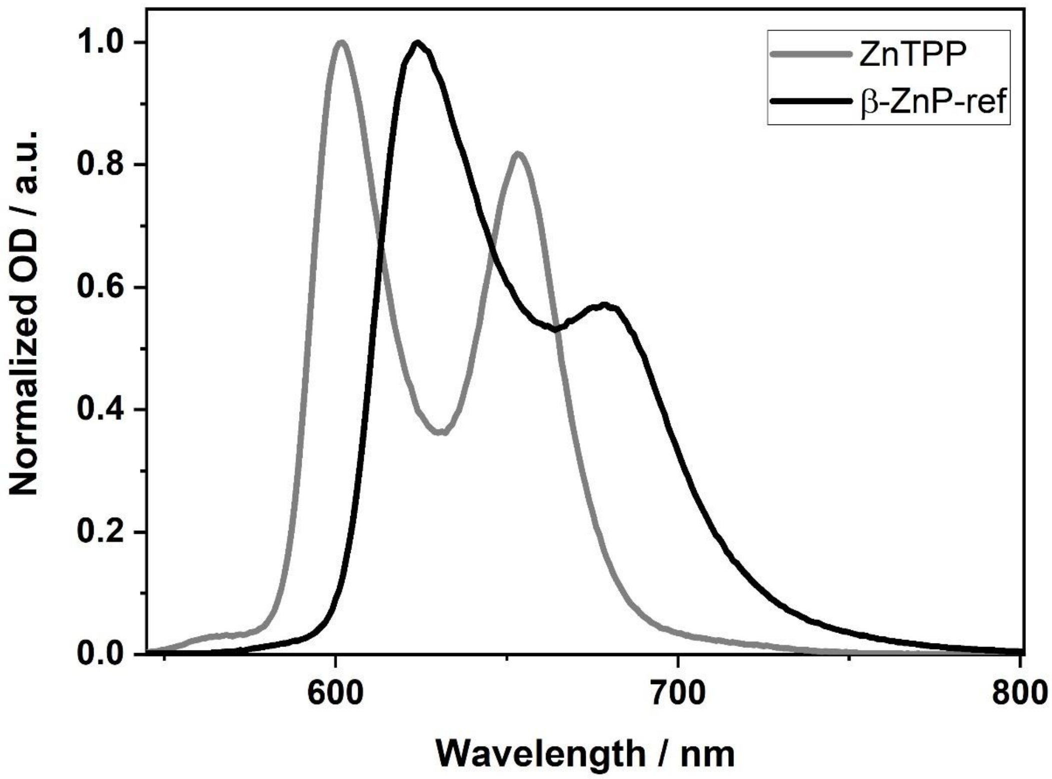

Figure S25. Fluorescence spectra of **ZnTPP** (grey) and **β-ZnP-ref** (black) recorded in THF at an excitation wavelength of 435 nm under ambient conditions.

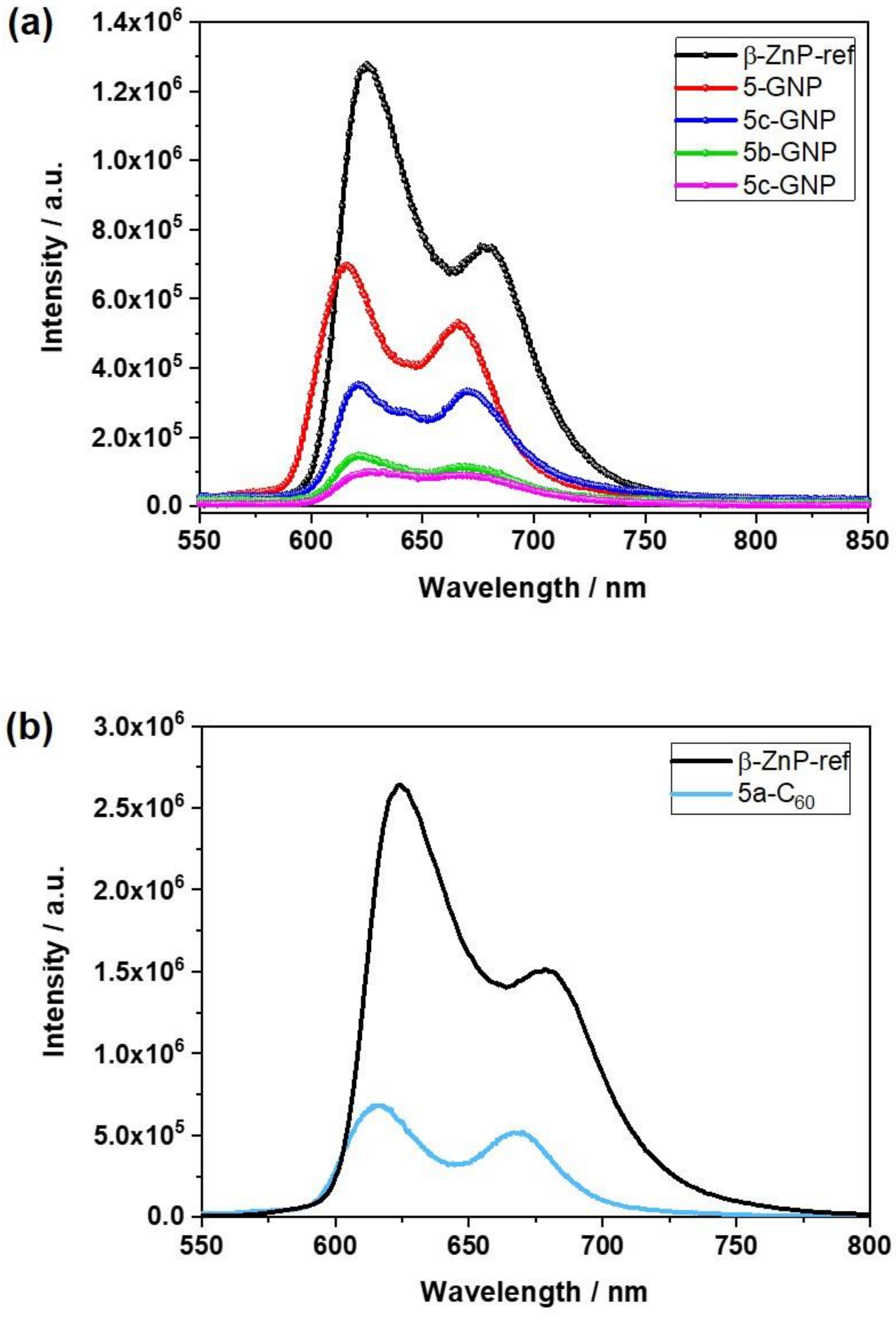

Figure S26. Fluorescence spectra **β-ZnP-ref** (black), **5-GNP** (red), **5a-GNP** (blue), **5-b-GNP** (green), and **5c-GNP** (magenta) recorded, (b): **β-ZnP-ref** (black), and **5a-C_{60}** (light blue), recorded in THF at an excitation wavelength of 435 nm under ambient conditions.

Stern Volmer Analysis

Conjugate	K_{sv}/[Q] from [I_0/I -1]	K_{sv}/[Q] from [τ_0/τ -1]
5-GNP	0.69	2.60
5a-GNP	8.14	10.84
5b-GNP	5.49	8.00
5c-GNP	1.66	2.27

Table S1: Values of the K_{sv}/[Q] reported using Stern-Volmer analysis for the conjugates. The values were determined from the fluorescence intensity ratio $\frac{I_{0\ \beta\text{-ZnP-ref}}}{I_{\text{conjugate}}}$, and the lifetime from the transient absorption measurements $\frac{\tau_{0\ \beta\text{-ZnP-ref}}}{\tau_{\text{conjugate}}}$., by using the β-ZnP-ref as reference for the properties without the quencher.

Electrochemical Studies

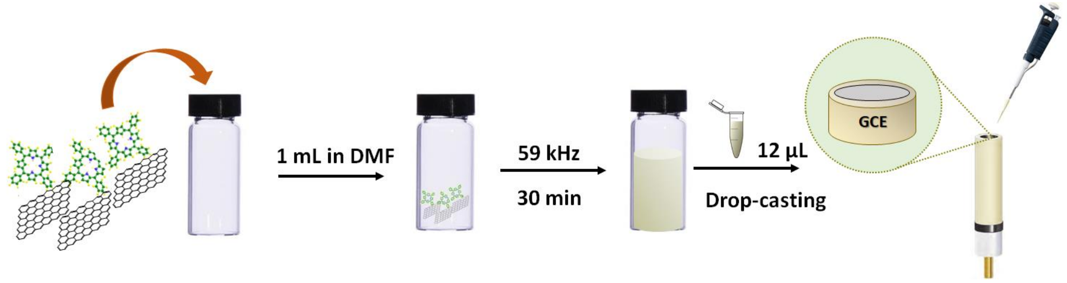

Scheme S1. Functionalization of the working electrode with the GNP-conjugates.

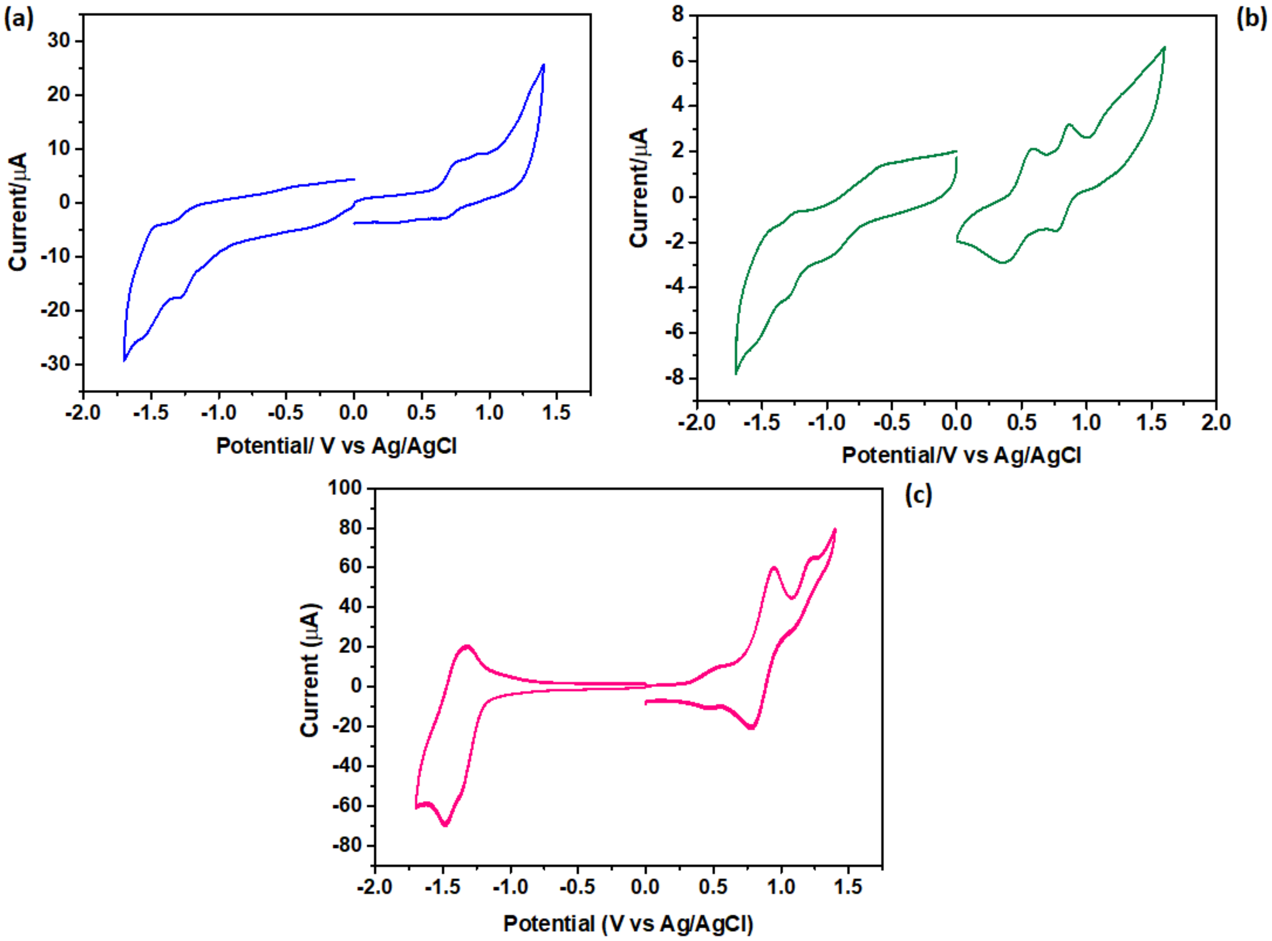

Figure S27. Cyclic voltammograms of **5a** (a), **5b** (b), and **5c** (c) in CH_3CN (0.1 M $TBAPF_6$) at room temperature using GC electrode and measuring at 100 mVs^{-1}.

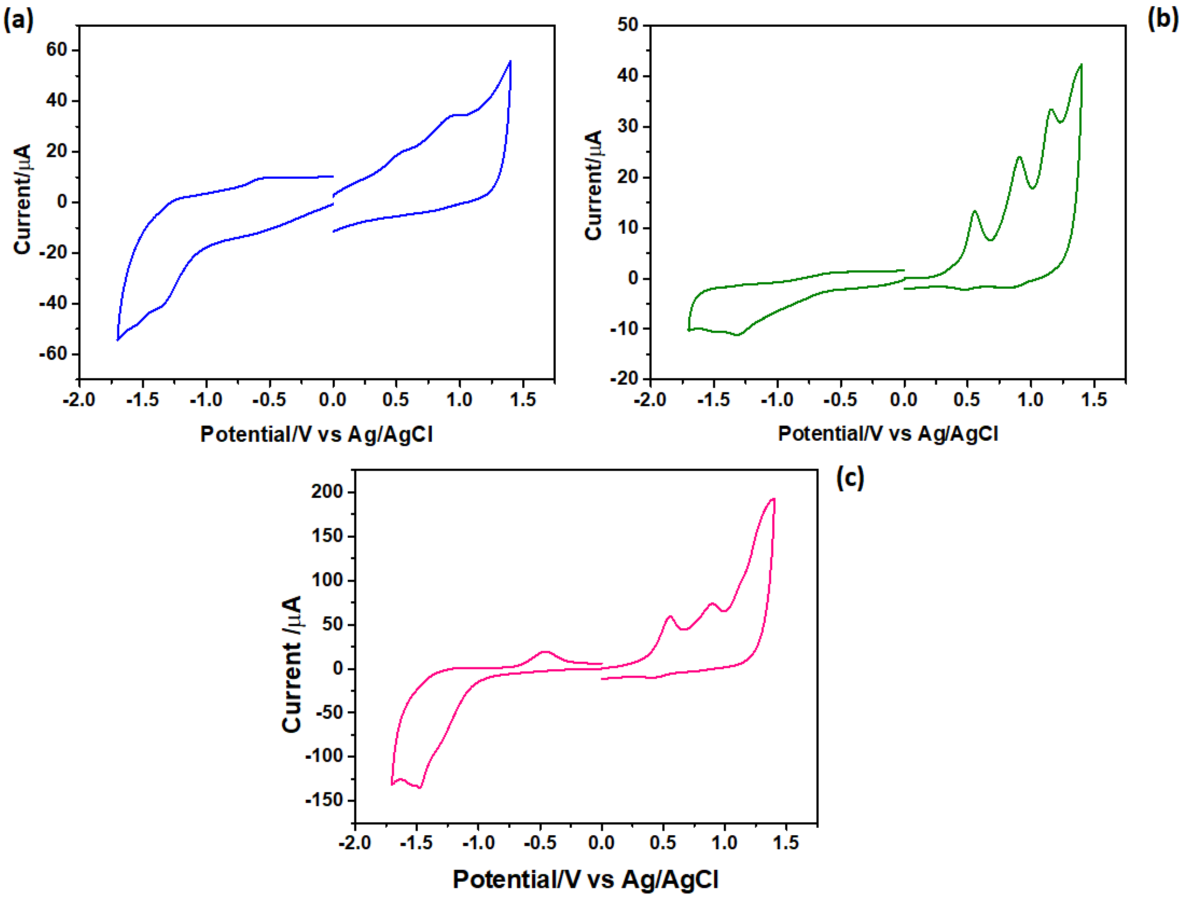

Figure S28. Cyclic voltammograms of **5a-GNP** (a), **5b-GNP** (b), and **5c-GNP** (c) in CH_3CN (0.1 M $TBAPF_6$) at room temperature using GC electrode and measuring at 100 mVs^{-1}.

Time-resolved absorption spectroscopy

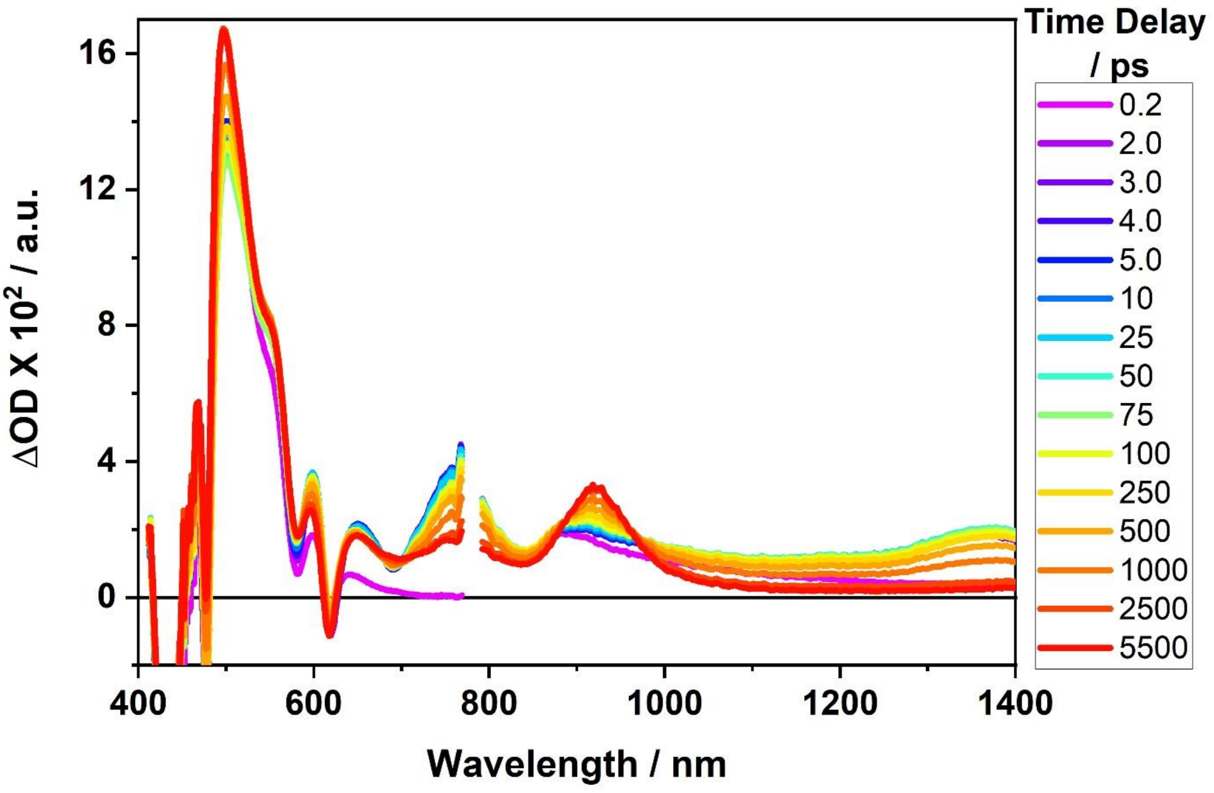

Figure S29. Fs-Transient absorption spectra of **β-ZnP-ref.** recorded at several time delays from 0.2 to 5500 ps in the visible and near-infrared regions at an excitation wavelength of 460 nm in THF under ambient conditions.

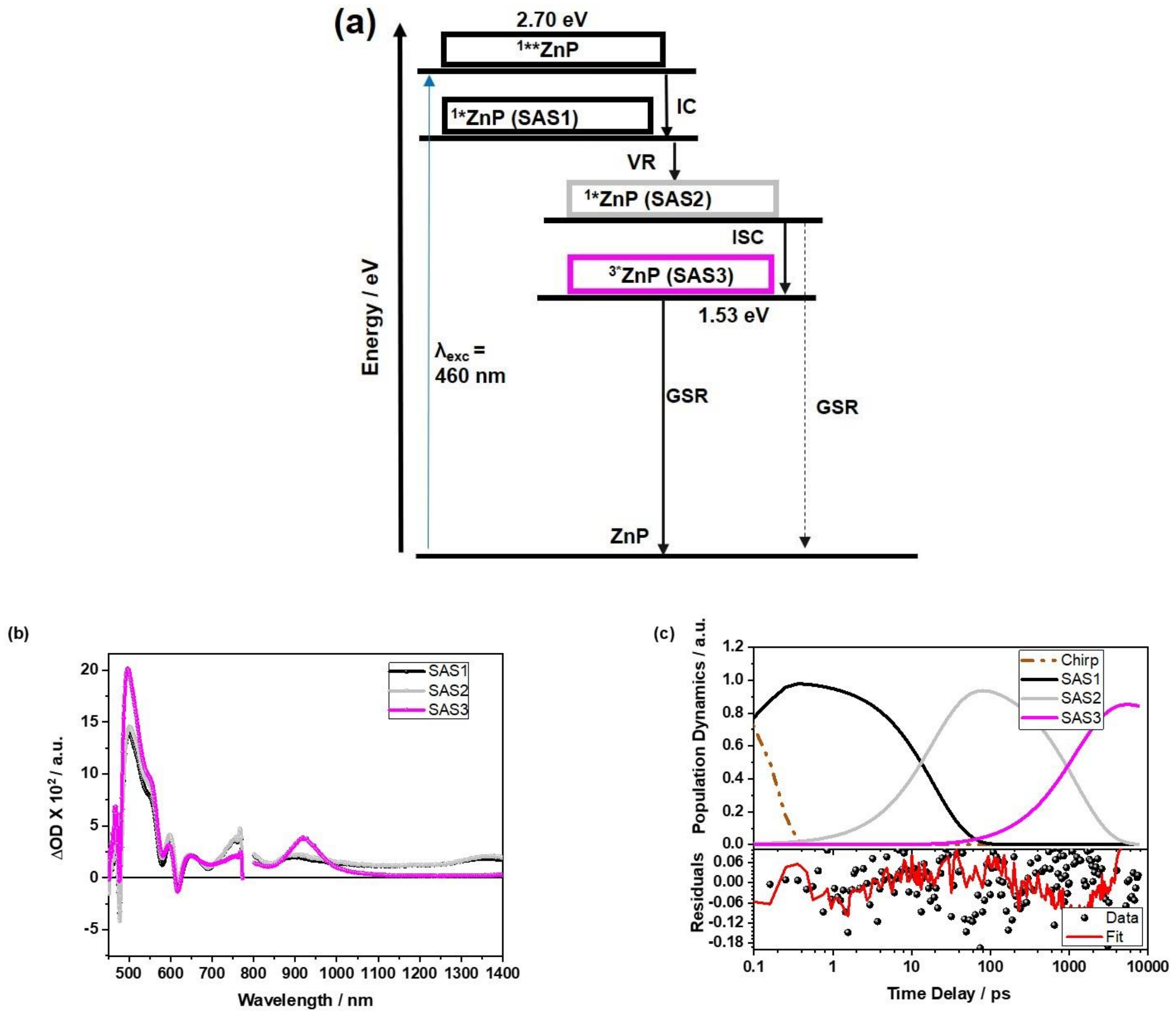

Figure S30. (a) The kinetic model applied to deconvolute the excited state species of **β-ZnP-ref** in THF via target analysis in GloTarAn. (IC: internal-conversion, VR: vibrational-relaxation, ISC: inter-system crossing, GSR: ground-state recovery). (b) Species associated spectra SAS of **β-ZnP-ref** with ^{1*}ZnP (SAS1 – black), vibrationally relaxed ^{1*}ZnP$_{rel}$ (SAS2 – gray), and ^{3*}ZnP (SAS3 – magenta) deconvoluted using the global-target analysis of the transient absorption data recorded at an excitation wavelength of 460 nm in THF under ambient conditions. (c) Population dynamics of the SAS1 (τ = 36.0 ps), SAS2 (τ = 1.0 ns), and SAS3 (τ > 10.0 ns), along with the residuals of the fits.

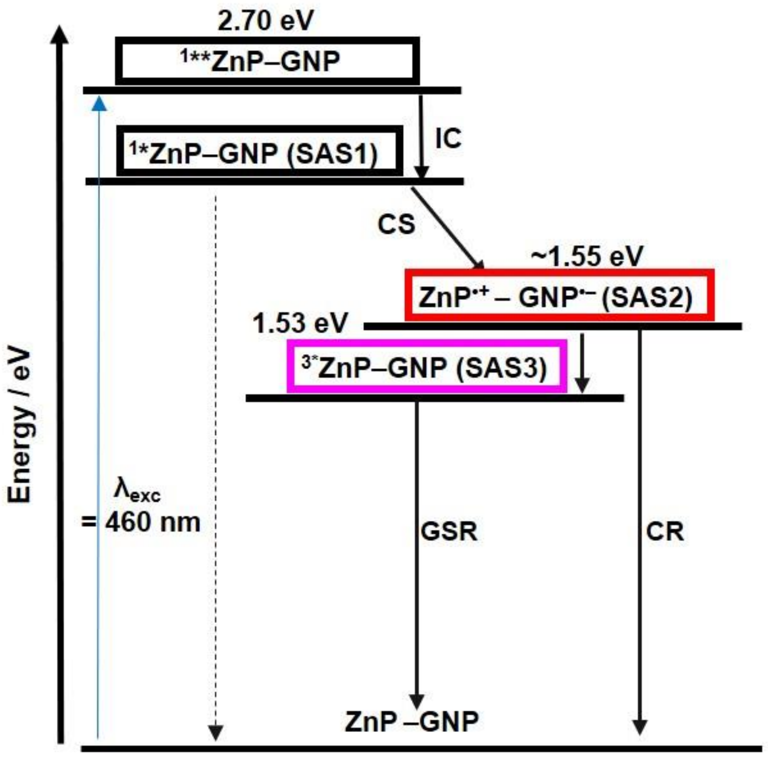

Figure S31. The kinetic model applied to deconvolute the excited state species of **5-GNP** in THF via target analysis in GloTarAn. (IC: internal-conversion, CS: charge-separation, CR: charge-recombination, and GSR: ground-state recovery).

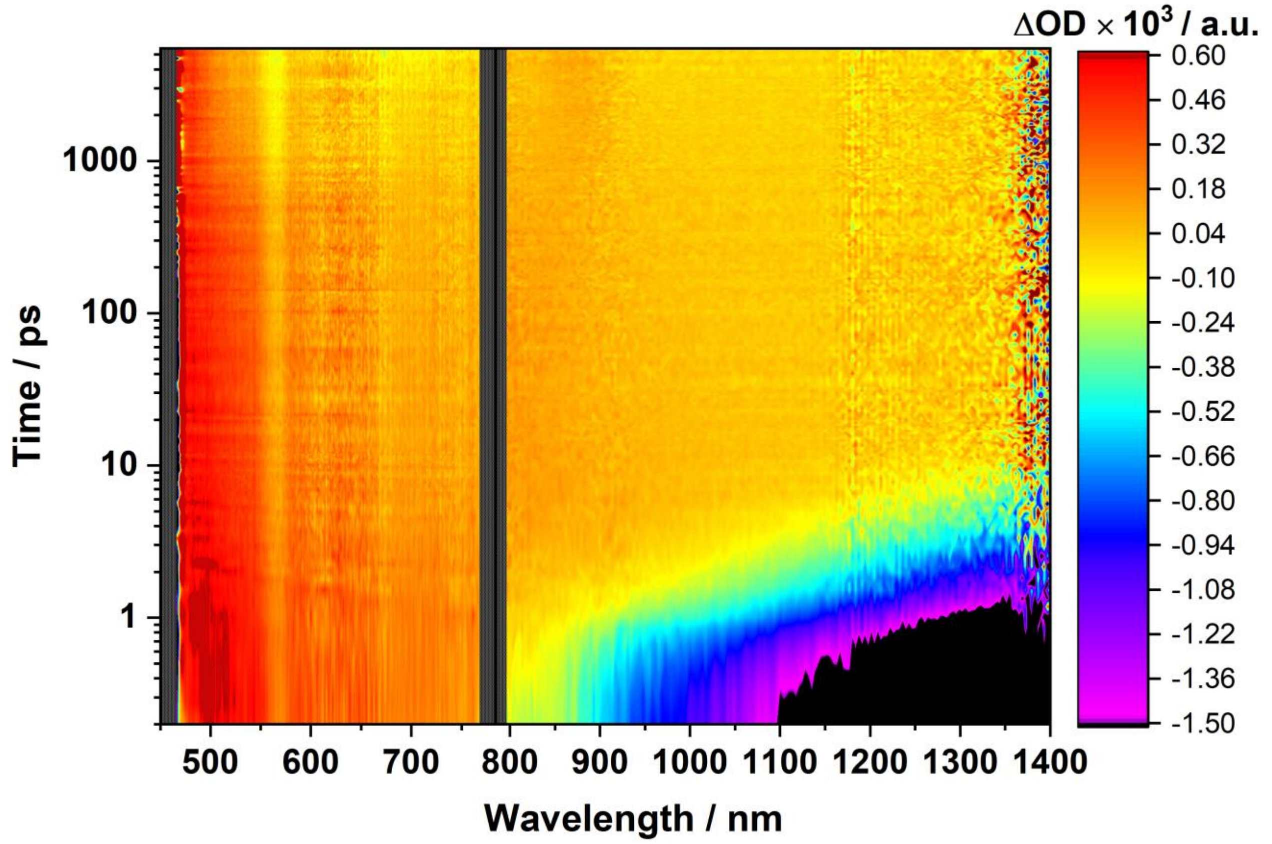

Figure S32. Fs-Transient absorption spectra of **5-GNP** recorded at several time delays from 0.2 to 5500 ps in the visible and near-infrared regions at an excitation wavelength of 460 nm in THF under ambient conditions.

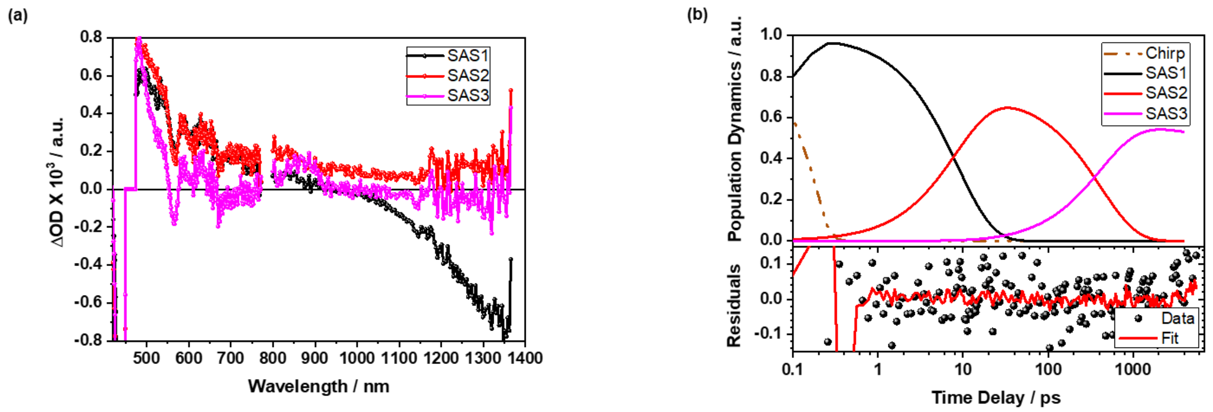

Figure S33. (a) Species associated spectra SAS of **5-GNP** with ^{1*}ZnP-GNP (SAS1 – black), $ZnP^{•+}$-$GNP^{•-}$ (SAS2 – red), and ^{3*}ZnP (SAS3 – magenta), deconvoluted using the global-target analysis of the transient absorption data recorded at an excitation wavelength of 460 nm in THF under ambient conditions. (b) Population dynamics of the SAS1 (τ = 9.90 ps; CS = 1.0 × 10^{11} s^{-1}), SAS2 (τ = 550 ps; CR into SAS3 = 1.3 × 10^{9} s^{-1} and CR into ground-state GS = 3.63 × 10^{8} s^{-1}), and SAS3 (τ > 10.0 ns), along with the residuals of the fits.

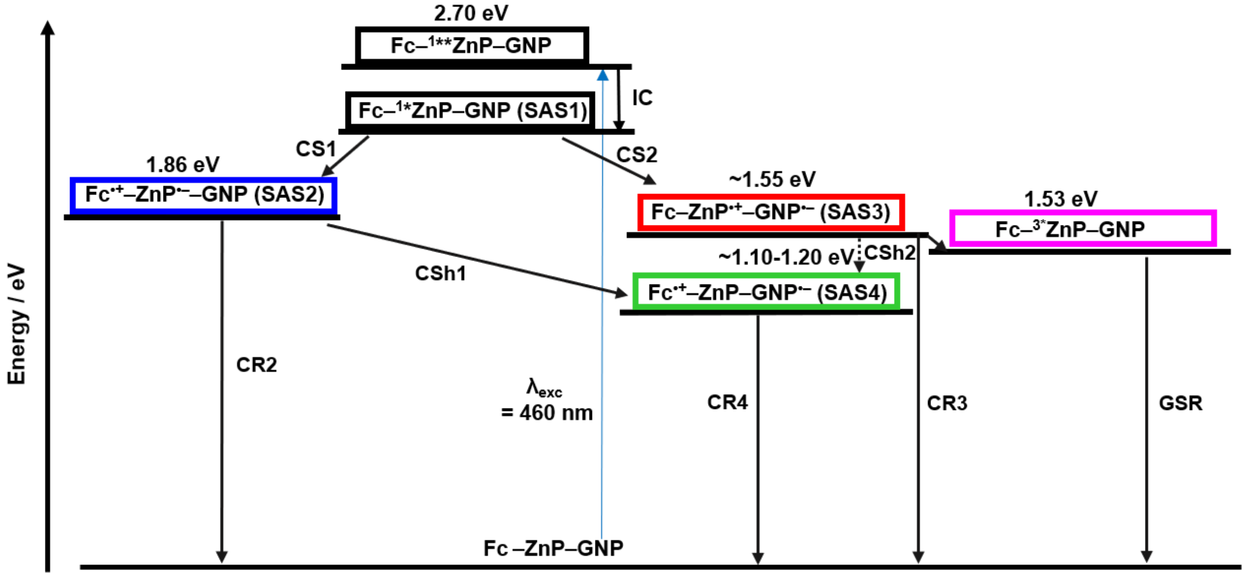

Figure S34. The kinetic model applied to deconvolute the excited state species of **5a-GNP** and **5b-GNP** in THF via global-target analysis in GloTarAn. (IC: internal-conversion, CS: charge-separation, CSh: charge-shift, and CR: charge-recombination, GSR = ground state recovery). ZnP/ZnP$^{•+}$ = 0.90 V,. ZnP/ZnP$^{•–}$ = 1.32 V, Fc/Fc$^{•+}$ = 0.54 V, and GNP/GNP$^{•–}$ = (−0.5) – (−0.8) V. The dashed arrow indicates that the process could not be deconvoluted.

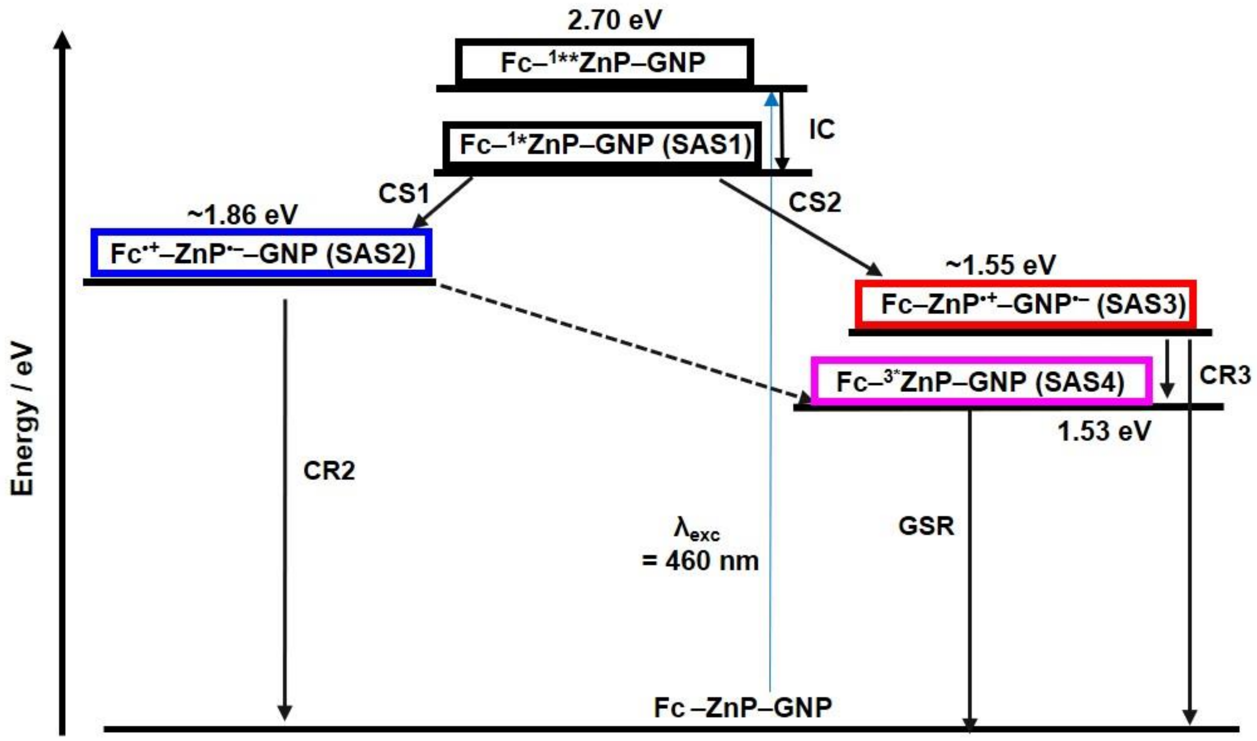

Figure S35. The kinetic model applied to deconvolute the excited state species of **5c-GNP** in THF via global-target analysis in GloTarAn. (IC: internal-conversion, CS: charge-separation, CSh: charge-shift, CR: charge-recombination, and GSR: ground-state recovery). ZnP/ZnP$^{•+}$ = 0.90 V, ZnP/ZnP$^{•–}$ = 1.32 V, Fc/Fc$^{•+}$ = 0.54 V, and GNP/GNP$^{•–}$ = (−0.5) – (−0.8) V. The dashed arrow indicates that the process could not be deconvoluted.

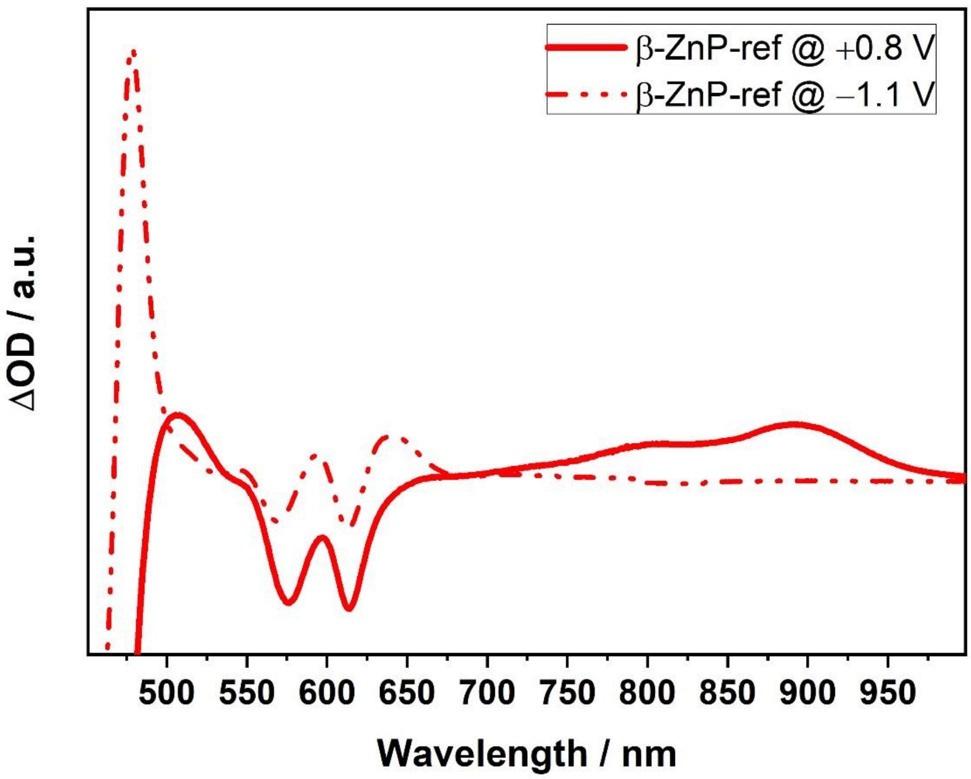

Figure S36. Differential absorption spectra of spectroelectrochemically oxidized (solid red) and reduced (dashed red) **β-ZnP-ref** recorded in argon-saturated THF with 0.1 M $TBAPF_6$ as the supporting electrolyte, Ag/Ag^+ as reference electrode, Pt as counter electrode, and Pt mesh as working electrode at an applied voltage of +0.8 and -1.1 V, respectively.

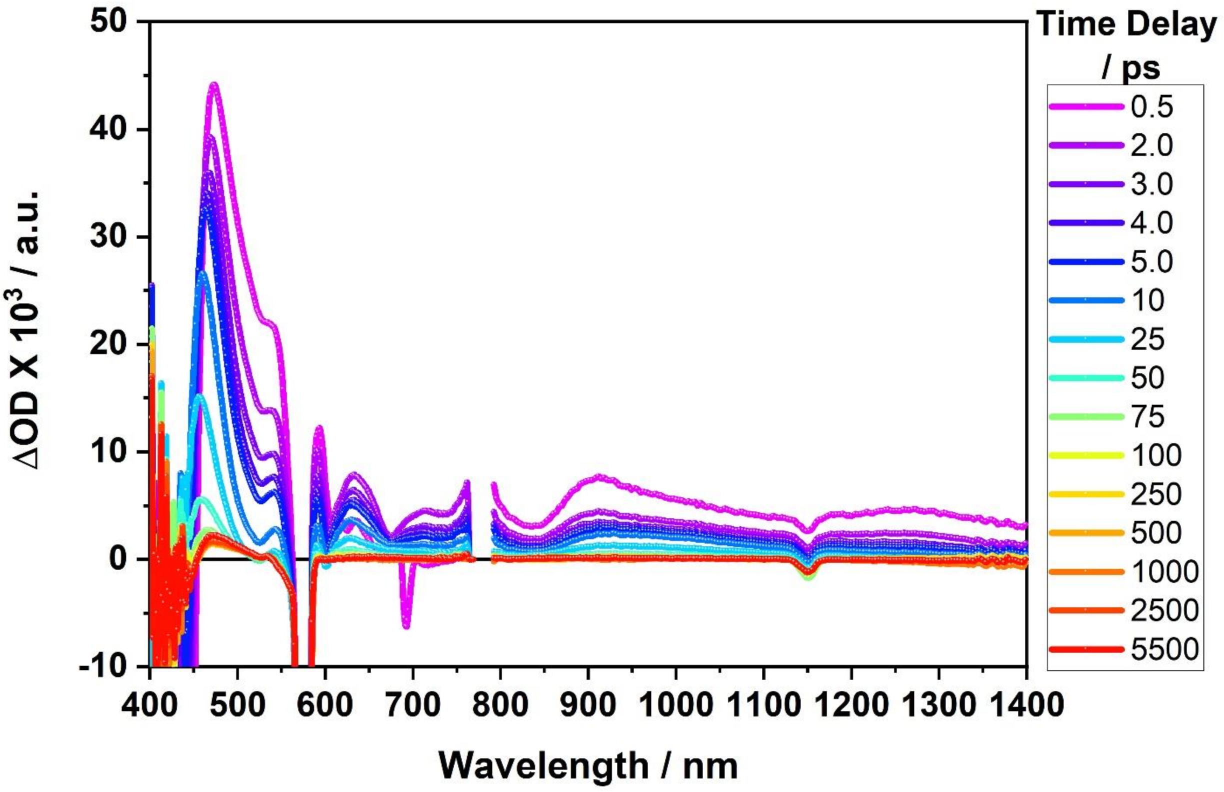

Figure S37. Fs-Transient absorption spectra of **HZnPa** recorded at several time delays from 0.5 to 5500 ps in the visible and near-infrared regions at an excitation wavelength of 568 nm in argon-saturated THF under ambient conditions.

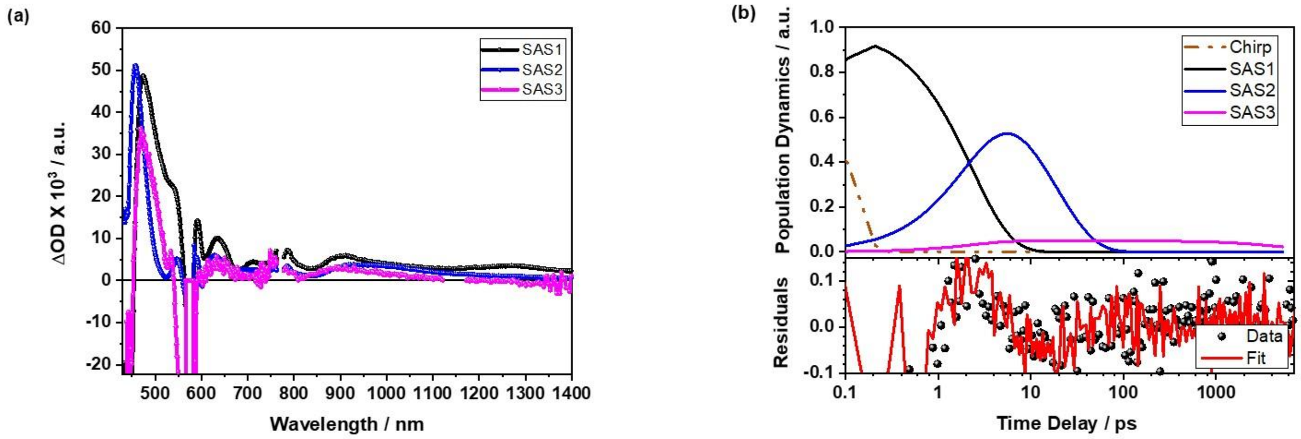

Figure S38. (a) Species associated spectra SAS of **HZnPa** with ^{1*}ZnP–Fc (SAS1 – black), ZnP$^{\bullet-}$–Fc$^{\bullet+}$ (SAS2 – blue), and long-lived >5ns (SAS3 – magenta), deconvoluted using the global-target analysis of the transient absorption data recorded at an excitation wavelength of 568 nm in argon-saturated THF under ambient conditions. (b) Population dynamics of the SAS1 (τ = 2.60 ps), SAS2 (τ = 22.2 ps), and SAS3 (τ > 5.0 ns), along with the residuals of the fits.

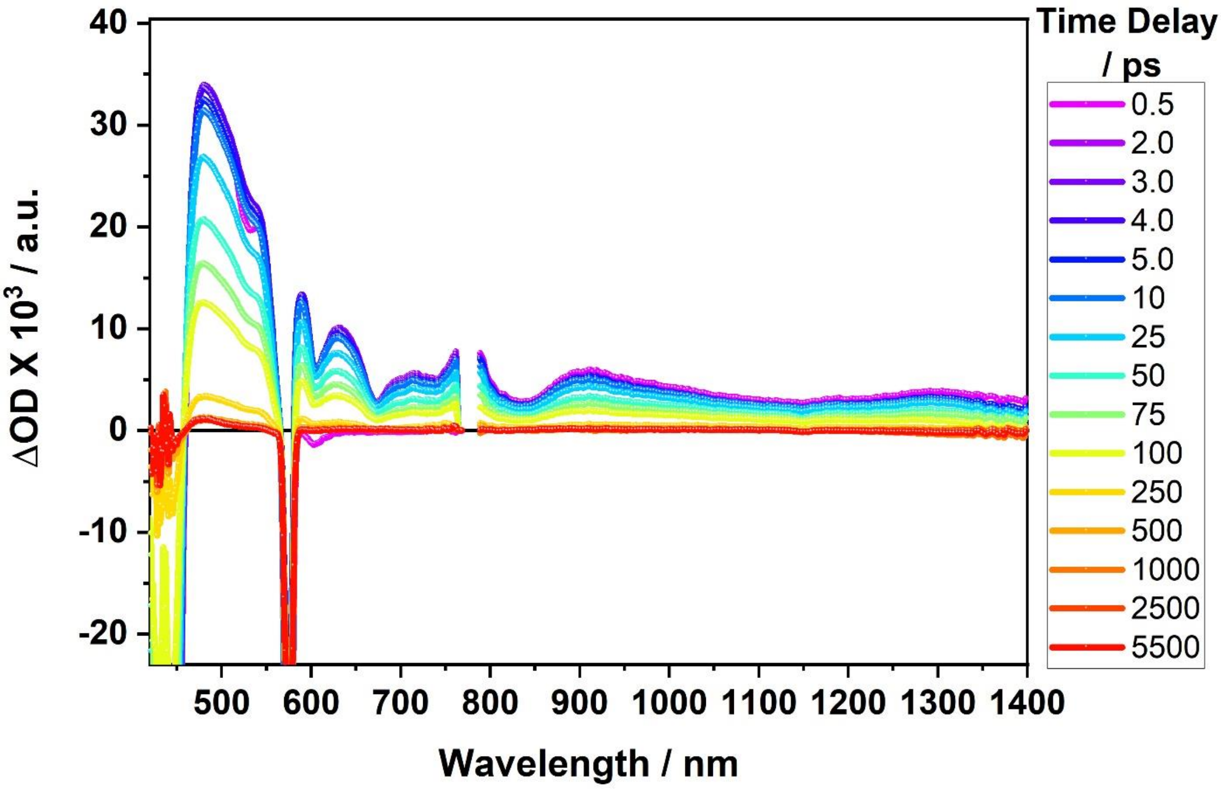

Figure S39. Fs-Transient absorption spectra of **HZnPb** recorded at several time delays from 0.5 to 5500 ps in the visible and near-infrared regions at an excitation wavelength of 568 nm in argon-saturated THF under ambient conditions.

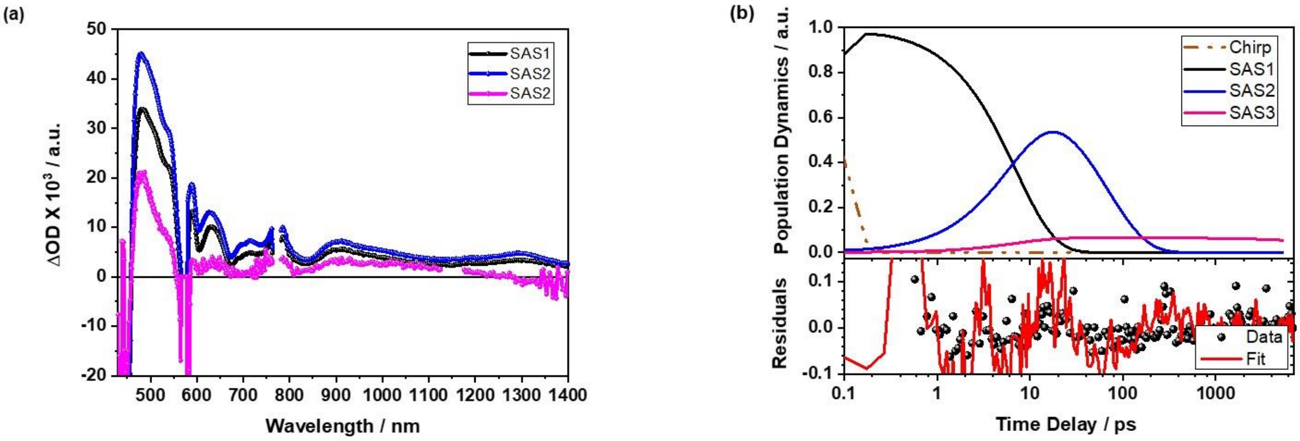

Figure S40. (a) Species associated spectra SAS of **HZnPb** with ^{1*}ZnP–Fc (SAS1 – black), ZnP$^{\bullet-}$–Fc$^{\bullet+}$ (SAS2 – blue), and long-lived >5ns (SAS3 – magenta), deconvoluted using the global-target analysis of the transient absorption data recorded at an excitation wavelength of 568 nm in argon-saturated THF under ambient conditions. (b) Population dynamics of the SAS1 (τ = 8.10 ps), SAS2 (τ = 85.2 ps), and SAS3 (τ > 5.0 ns) along with the residuals of the fits.

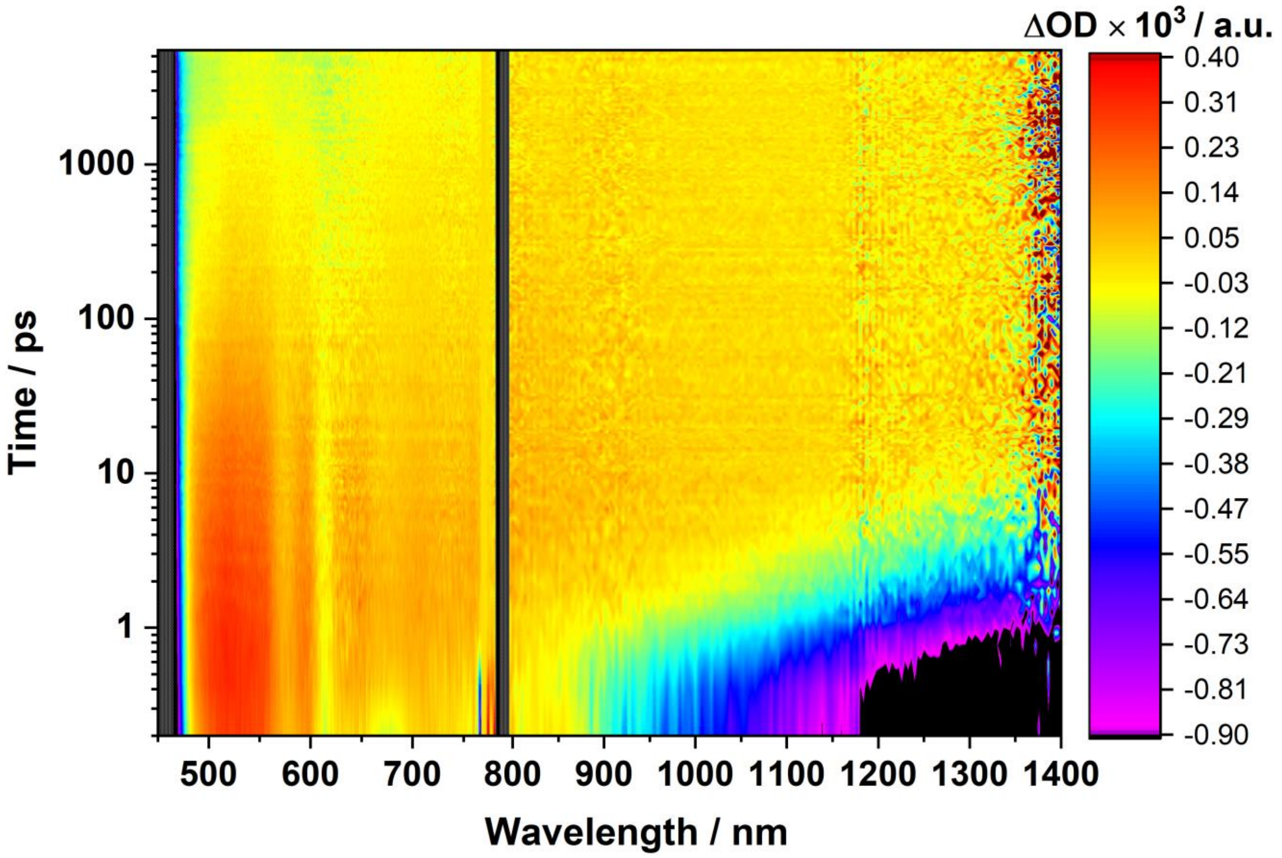

Figure S41. Fs-Transient absorption spectra of the **5b-GNP** recorded at several time delays from 0.2 to 5500 ps in the visible and near-infrared regions at an excitation wavelength of 460 nm in THF under ambient conditions.

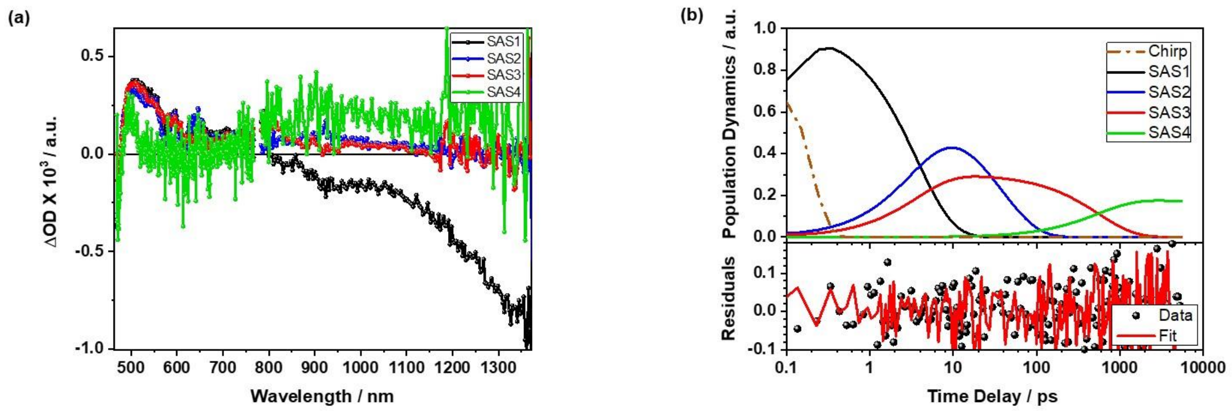

Figure S42. (a) Species associated spectra SAS of the **5b-GNP** with Fc-^{1*}ZnP-GNP (SAS1 – black), $Fc^{\bullet+}$-$ZnP^{\bullet-}$-GNP (SAS2 – blue), Fc-$ZnP^{\bullet+}$-$GNP^{\bullet-}$ (SAS3 – red), and ^{3*}ZnP/$Fc^{\bullet+}$-ZnP-$GNP^{\bullet-}$ (SAS4 – green), deconvoluted using the global-target analysis of the transient absorption data recorded at an excitation wavelength of 460 nm in THF under ambient conditions. (b) Population dynamics of the SAS1 (τ = 4.0 ps; CS1 = 1.2 × 10^{11} s^{-1} and CS2 = 1 × 10^{11} s^{-1}), SAS2 (τ = 38.5 ps; CR2 = 1.56 × 10^{10} s^{-1} and CSh1 = 1.04 × 10^{10} s^{-1}), SAS3 (τ = 550 ps; CR3 into GS = 6.80 × 10^8 s^{-1}), and SAS4 (τ > 10.0 ns), along with the residuals of the fits.

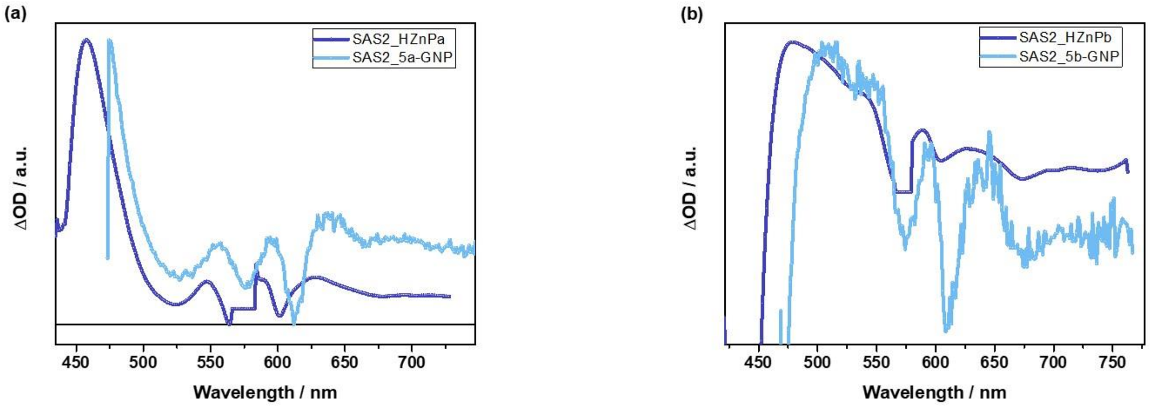

Figure S43. (a) Differential absorption spectra of the Fc$^{\bullet+}$-ZnP$^{\bullet-}$-GNP species of **5a-GNP** (SAS2 – light blue) compared to the Fc$^{\bullet+}$-ZnP$^{\bullet-}$ species of **HZnPa** (SAS2 – blue). (b) Differential absorption spectra of the Fc$^{\bullet+}$-ZnP$^{\bullet-}$-GNP species of **5b-GNP** (SAS2 – light blue) compared to the Fc$^{\bullet+}$-ZnP$^{\bullet-}$ species of **HZnPb** (SAS2 – blue) in order to verify the formation of the ZnP$^{\bullet-}$ which shares resemblance in the characteristic of the features observed.

[1] H.-C. Hsu , I. Shown, H.-Y. Wei, Y.-C.Chang, H.-Y. Du, Y.-G. Lin, C. -A. Tseng, C.-H. Wang, L.-C. Chen, Y-C. Lin, K.-H. Chen, *Nanoscale* **2013**, *5*, 262–268.

[2] M. H. Gehlen, *J. Photochem. Photobiol. C* **2020**, 42, 100338-100352.
